# Contrasting levels of transcriptome-wide SNP diversity and adaptive molecular variation among conifers

**DOI:** 10.3389/fpls.2025.1500759

**Published:** 2025-03-06

**Authors:** Nathalie Pavy, Sébastien Gérardi, Julien Prunier, Philippe Rigault, Jérôme Laroche, Gaétan Daigle, Brian Boyle, John MacKay, Jean Bousquet

**Affiliations:** ^1^ Canada Research Chair in Forest Genomics, Institute for Systems and Integrative Biology and Forest Research Centre, Université Laval, Québec, QC, Canada; ^2^ Institute for Systems and Integrative Biology, Université Laval, Québec, QC, Canada; ^3^ Département de Médecine Moléculaire, Faculté de Médecine, Université Laval, Québec, QC, Canada; ^4^ Gydle Inc., Québec, QC, Canada; ^5^ Département de Mathématiques et de Statistiques, Faculté des Sciences et de Génie, Université Laval, Québec, QC, Canada; ^6^ Department of Biology, University of Oxford, Oxford, United Kingdom

**Keywords:** intraspecific molecular genetic diversity, molecular and functional adaptive variation, SNP A/S ratio, comparative genomics, expressed genes, Cupressaceae, Pinaceae, positively selected genes

## Abstract

Adaptive convergence can arise when response to natural selection involves shared molecular or functional mechanisms among multiple taxa. Conifers are archaic species of ancient origin with delayed sexual maturity related to their woody perennial nature. Thus, they represent a relevant plant group to assess if convergence from selection may have become disconnected between molecular and functional levels. In this purpose, transcriptome-wide SNP diversity was assessed in seven partially sympatric and reproductively isolated conifer species (118 individuals from 67 populations) populating the temperate and boreal forests of northeastern North America. SNP diversity was found highly heterogeneous among species, which would relate to variation in species-specific demography and history. Rapidly evolving genes with signatures of positive selection were identified, and their relative abundance among species reflected differences in transcriptome-wide SNP diversity. The analysis of sequence homology also revealed very limited convergence among taxa in spite of sampling same tissues at same age. However, convergence increased gradually at the levels of gene families and biological processes, which were largely related to stress response and regulatory mechanisms in all species. Given their multiple small to large gene families and long time since inception, conifers may have had sufficient gene network flexibility and gene functional redundancy for evolving alternative adaptive genes for similar metabolic responses to environmental selection pressures. Despite a long divergence time of ~350 Mya between conifers and Angiosperms, we also uncovered a set of 17 key genes presumably under positive selection in both lineages.

## Introduction

Adaptive genetic variation allows organisms to cope with natural selective pressures and thrive in their environment. This is especially true for long-lived woody plants, such as conifers from mid-northern latitudes, that must contend with delayed sexual maturity to adapt to highly heterogeneous and changing climatic conditions (Depardieu et al., 2021). Therefore, identifying and characterizing adaptive genetic variation within species is crucial to understand the molecular mechanisms underlying their response to environmental pressures. Molecular convergence can arise when such molecular mechanisms are shared by multiple species (Stern, 2013). This process may occur at different hierarchical levels, such as specific nucleotides, protein-coding genes (often referred to as ‘gene reuse’), gene families, or genes belonging to the same biological pathways (Hao et al., 2019; Sackton and Clark, 2019). As a general trend, molecular convergence is expected to increase with hierarchical levels under similar positive selection pressures (Stern, 2013; He et al., 2020; Xu et al., 2020) in spite of divergent adaptive evolution at the gene level.

However, the many determinants of molecular convergence complicate the prediction of patterns of adaptive evolution at both intraspecific and interspecific taxonomic levels. The most influential determinants include ancestry (the probability of convergence decreases along with taxa divergence time), effective population size (taxa of small effective population size are less likely to converge due to increased genetic drift), gene flow/introgression (gene flow usually increases convergence by constraining differentiation among taxa, but can also prevent or delay local adaptation), selection landscape (convergence is expected to decrease when the number of selective pressures increases in a given habitat), and many-to-one mapping (convergence is expected to decrease as the number of traits governing a given functional output increases) (reviewed by Bolnick et al., 2018).

Many-to-one mapping is a particularly relevant determinant of molecular convergence when studying the adaptive trajectories of lineages and species. Indeed, considering that the link between phenotypic and molecular convergence is well established in a variety of taxa (see Martin and Orgogozo, 2013 for a catalog of genetic hotspots of phenotypic variation in animals, plants, and yeast), it is reasonable to assume that molecular convergence reflects shared adaptive response to similar selective pressures. However, the opposite is not necessarily true. The fact that adaptative traits are usually highly polygenic (e.g. Le Corre and Kremer, 2012; Csilléry et al., 2018; Barghi et al., 2020; Depardieu et al., 2021) suggests that plant taxa have typically many genetic solutions available to solve the adaptive challenges they face in nature (Arendt and Reznick, 2008; Losos, 2011; Tenaillon et al., 2012; Storz, 2016), including in closely-related populations from the same species (e.g. Manceau et al., 2010; Elmer and Meyer, 2011). Most plant groups such as conifers are also characterized by large gene families and redundancy of gene function (Guillet-Claude et al., 2004; Bedon et al., 2010; Pavy et al., 2012a; Stival Sena et al., 2018; Van Ghelder et al., 2019). Therefore, lineages and species may follow similar adaptive trajectories, while showing reduced levels convergence at the molecular level. Hence, assessing the extent of functional convergence of adaptive genes in multiple lineages and species can complement the picture derived from molecular convergence alone, and reveal otherwise hidden adaptive patterns.

To address these fundamental questions related to adaptive convergence, conifers from northeastern North America represent an ideal framework for several reasons. First, contrary to European forests for instance, these forests have been generally characterized by low levels of anthropic disturbance up to the twentieth century (i.e. reduced urbanization and forest management) and the regional landscape is of relative topographic homogeneity, thus facilitating gene flow, compared to western North America for instance. The most significant barriers to gene flow in eastern North America include the Great Lakes and the Appalachian Mountains, which are thought to be responsible for the genetic divergence of most historical lineages still observable nowadays in eastern North American conifers (Jaramillo-Correa et al., 2009). The two main glacial refugia in this region would have been located south of the Great Lakes and east of the Appalachian Mountains along the Atlantic coast (Jaramillo-Correa et al., 2009). With the limited potential of confounding factors from long-term human activity and the lack of significant barriers constraining migration during the Holocene, tree species could track their most suitable current habitats and evolve local adaptations in response to environmental selective pressures, as evidenced by several empirical studies (e.g. Namroud et al., 2008; Prunier et al., 2011; Hornoy et al., 2015; Nadeau et al., 2016). Second, none of the conifers in the boreal forest of northeastern North America are known to hybridize, although they are sympatric in most of their range: potential hybrid zones are all located at the southern or western edge of the species ranges, and they have been quite well delimited and were therefore easy to avoid by using an adequate sampling strategy (Jaramillo-Correa et al., 2009), which would minimize the risk that molecular signatures of natural selection within species are confounded by interspecific introgression. Also, the extensive range overlap of conifer species across the mid-latitude forests of northeastern North America indicates that these species generally face common environmental pressures, of which harsh and heterogenous climatic conditions are a large component (Hornoy et al., 2015; Depardieu et al., 2021). Thus, these conifers represent relevant models to address questions about long-term evolution and adaptation from a comparative perspective.

However, studying molecular convergence in woody perennial plant taxa with such large and complex genomes is highly challenging. Over the last 20 years, our understanding of conifer genomes has progressed significantly through the sequencing and analyses of their genome structure, evolution and functions (De La Torre et al., 2014; Prunier et al., 2016). Nonetheless, extensive resequencing has been restricted to only a few conifer species belonging primarily to the *Picea* and *Pinus* genera. This limits the potential to conduct exhaustive comparative studies across conifers, which are essential to understand the common determinants of adaptive evolution. To date, the main findings indicate a rather limited convergence among adaptive genes identified from species belonging to the same or different genera (Mosca et al., 2012; Yeaman et al., 2016; Bousquet et al., 2021; Gagalova et al., 2022).

In this study, we investigated adaptive molecular convergence at the transcriptome-wide level among six sympatric Pinaceae species native of northeastern North America, namely white spruce (*Picea glauca*), black spruce (*Picea mariana*), eastern white pine (*Pinus strobus*), jack pine (*Pinus banksiana*), balsam fir (*Abies balsamea*), and tamarack (*Larix laricina*), as well as one sympatric Cupressaceae taxon, eastern white cedar (*Thuja occidentalis*), for a total of 118 individuals representing 67 populations. Considering that the Cupressaceae and the Pinaceae diverged ~315 Mya (Leslie et al., 2018), while taxa divergence within the Pinaceae did not take place before ~185 Mya (divergence of the *Abies* genus from its sister taxa; Leslie et al., 2018), we included a Cupressaceae taxon to qualitatively assess the effect of phylogenetic relatedness on our inferences. In this study, we first identified gene nucleotide polymorphisms within each species, and assessed their level of overall genetic diversity across much of the transcriptome. We then identified genes with sequence signatures of positive selection in each species in order to estimate the level of adaptive convergence among species from a molecular and functional perspectives. This approach also allowed us to identify shared drivers of adaptive molecular evolution among species. We also investigated the extent of adaptive molecular convergence between Angiosperms and this group of conifers despite their ancient phylogenetic divergence (Savard et al., 1994).

## Materials and methods

### Biological materials

**Figure 1:**
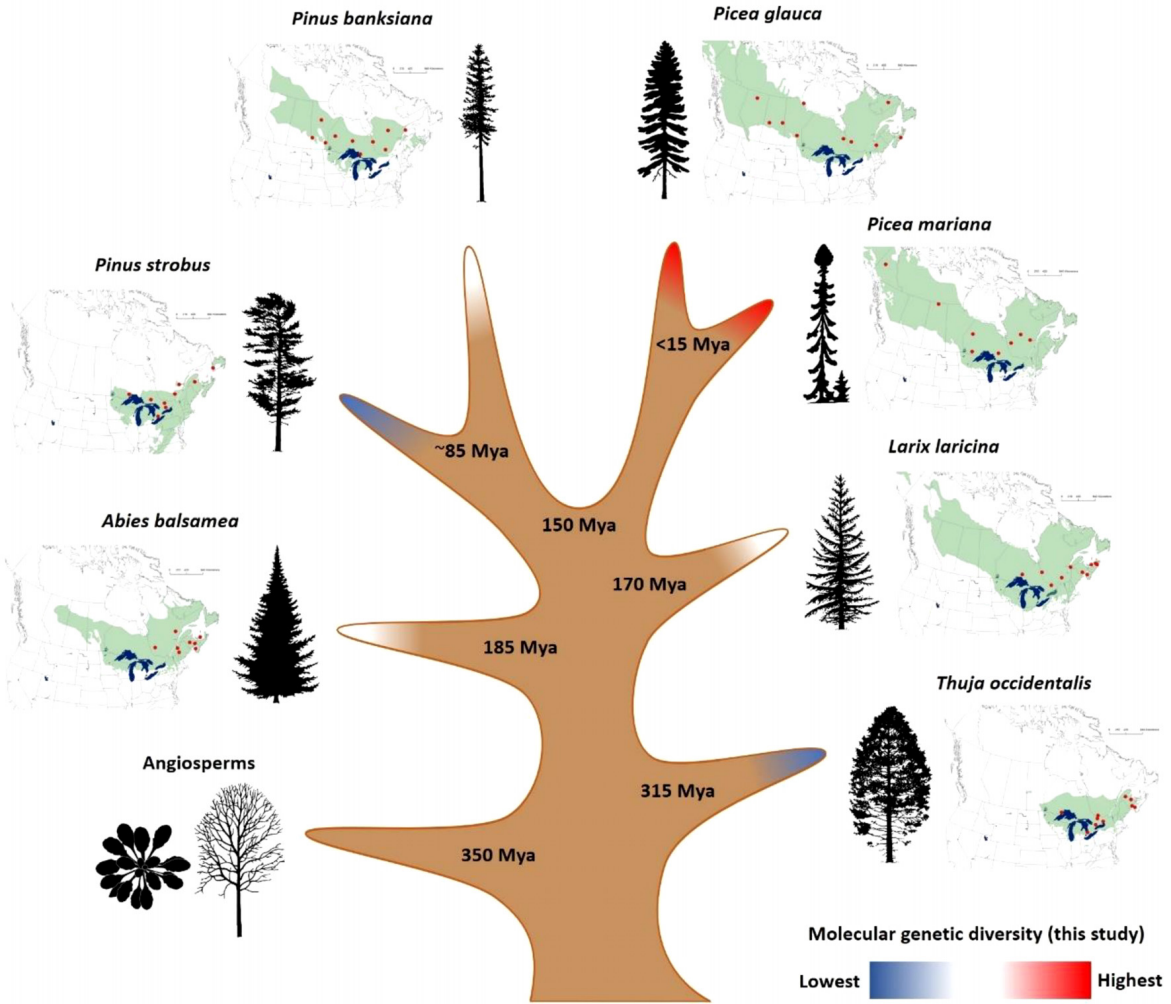
Characteristics of the seven conifer species analyzed in this study. The approximate natural range of each species is represented in green besides its associated tree silhouette, and populations sampled are mapped as red dots. For illustrative purposes only, the figure shows species divergence times derived from fossil-calibrated molecular clocks (Bouillé and Bousquet, 2005; Leslie et al., 2018; Li et al., 2019). Colors at branch tips represent the three groups of overall SNP diversity identified in this study at the transcriptome-wide level (see Results). Source of tree silhouettes: https://tidcf.nrcan.gc.ca/; https://www.phylopic.org/.

Seven conifer species were sampled, namely *Picea glauca*, *Picea mariana*, *Pinus strobus*, *Pinus banksiana*, *Abies balsamea*, *Larix laricina*, and *Thuja occidentalis*. For each species, seeds from ten provenances were obtained from the National Tree Seed Center (Fredericton, New-Brunswick, Canada), paying special attention to avoid provenances located within sympatric or paratric zones in species known to spontaneously hybridize with related taxa (**Figure 1**; **Supplementary Methods S1**). For each species, between 15 and 18 diploid embryos representing ten distinct provenances (two seeds per provenance on average) were extracted and flash frozen in separate tubes. All species considered, a total of 118 individuals from 67 populations were sequenced. In addition, four provenances were randomly selected per species and one seed per provenance was dissected to extract the haploid megagametophyte, which was flash frozen in liquid nitrogen in separate tubes. Megagametophytes were barcoded individually, while embryos were pooled at equimolar concentration and barcoded as a single library, prior to the sequencing step (**Supplementary Methods S1**). All SNPs identified subsequently in the pool of embryos and in at least one megagametophyte were discarded so to filter out paralogous non-mendelian SNPs (see next section 'SNP calling').

### Sequencing

Total RNA was extracted using the MasterPure™ Plant RNA Purification kit (Epicenter, Madison, WI, USA). RNAs were sequenced in paired-end mode (2×125 bp) with an Illumina HiSeq 2500 (Methods S2). Raw sequencing data (reads) were deposited in the public database ENA (European Nucleotide Archive, https://www.ebi.ac.uk/ena/browser/home, accessions ERS16017105-ERS16017139 and ERS16049778-ERS16049791) and vcf files containing variants identified in each species were deposited in DRYAD ('https://doi.org/10.5061/dryad.p8cz8w9w1). We assessed the good representativity of the analyzed transcriptomes based on sequence similarity searches (Methods S3).

### SNP calling

After sequence quality controls and filtering (**Supplementary Methods S4**), reads were aligned to the reference transcriptomes of each species previously published (Van Ghelder et al., 2019). SNPs were called using HaplotypeCaller version 3.4, from the GATK tool kit (McKenna et al., 2010; DePristo et al., 2011) and subsequently quality-filtered (**Supplementary Methods S5**). Since the megagametophyte is a haploid tissue in all seven conifer species investigated herein, SNPs identified within megagametophyte libraries were likely indicative of variations between paralogous gene sequences also occurring in embryos. Thus, these SNPs were considered as false-positives and were subtracted from SNPs identified in pools of embryos before subsequent analyses were carried out. In total, above 1.4 million raw SNPs were identified (**Supplementary Methods S5**, **Supplementary Table S5.1**). After removal of paralogous SNPs identified in haploid megagametophytes, around 867K SNPs remained (**Supplementary Table S5.1**). Among them, ~398K high-quality SNPs (**Supplementary Methods S5**), were located in coding sequences representing almost ~97K Open Reading Frames in total (~16K transcripts per species on average) (**Supplementary Table S1**). All species considered and on average, ~82% of the transcripts and ~70% of the ORFs carried SNPs (**Supplementary Table S1**).

Transcripts that were included in the comparison of molecular genetic diversity across species and the analysis of the functional annotations of polymorphic gene sequences had to exhibit an average coverage of 10 reads or more, at least 300 nucleotides with a depth of 10 or more reads, and contain coding sequences.

### SNP abundance in transcripts and comparison across species

The length and read depth of transcripts were significantly heterogenous across the seven conifer species investigated (**Supplementary Methods S6**). It is essential to control for such effects before analyzing SNP abundance differences across species (Eo and DeWoody, 2012). In this purpose, we applied a regression model assuming that the number of SNPs within transcripts follows a negative binomial distribution (Eo and DeWoody, 2012).

The model corrected efficiently for variations among transcripts depth and length, thus enabling a more rigorous comparison of the SNP abundance across species (**Supplementary Methods S7**). Then, SNP rate heterogeneity among species was tested using a Kruskall-Wallis test. To group species based on their level of total SNP diversity, Kolmogorov-Smirnov and Cramer-von Mises tests were conducted.

### Estimation of gene SNP A/S ratios to identify positively selected genes (PSGs)

Based on the premise that nonsynonymous substitutions are predicted to contribute more to adaptive evolution than synonymous substitutions (Stern and Orgogozo, 2008), one way to study molecular convergence is to compare the ratio of substitution rates at nonsynonymous (*K*a) versus synonymous (*K*s) sites in orthologous protein-coding sequences betwen species. Similar inferences can be drawn within taxa from gene SNP A/S ratios, since both ratios have been shown to be strongly positively correlated (Liu et al., 2008).

The SNP A/S ratio was then calculated for each gene as the number of SNPs per nonsynonymous site (A) divided by the number of SNPs per synonymous site (S). An adjusted SNP A/S ratio was used to include genes with no synonymous SNPs following the empirical logit principle (Agresti, 2013):


$$Adj.\;SNP\;A/S\;ratio=\frac{\left(number\;of\;nonsynonymous\;SNPs\;+\;0.5)/(La+1\right)}{(number\;of\;synonymous\;SNPs+0.5)/(Ls+1)\;}$$

The SNP A/S ratio was calculated over the longest open reading frame predicted for each transcript in each species. In order to identify putative positively selected genes (PSGs), we first retained those with A/S values exceeding 1, the threshold usually considered as evidence for positive selection (Kimura, 1983), and applied a subsequent filtering step to ensure that the probability that a transcript has an A/S ratios exceeding 1 by chance alone was lower than 5%, thus reducing much the number of false positives (see in **Supplementary Methods S8**). The resulting sets of PSGs were then used to analyze and compare the sequences and functional annotations of these deemed PSGs across the seven conifer species considered in the study.

### Sequence annotation and analyses

Predicted protein sequences were clustered into orthogroups with OrthoFinder version 2.3.8 (Emms and Kelly, 2019) run with default settings. Functional annotations of ORFs were derived from sequence similarity searches conducted with blastp version 2.13.0 against Uniprot (E-value <e^-15^) and PFAM (El-Gebali et al., 2019). Sequences were also assigned to Gene Ontology (GO) classes by using the mapping between the UniprotKB sequences and the GO terms. The heatmaps were generated using the pheatmap R package (Kolde, 2019).

Enrichment tests were conducted with the R (version 4.0.2) package topGO (version 2.42.0; Alexa et al., 2006; https://bioconductor.org/packages/release/bioc/html/topGO.html), in order to identify GO terms enriched among annotations of the genes with high SNP A/S values (**Supplementary Methods S9**).

### Sequence comparisons with positively selected genes in Brassica or poplar

PSGs were identified in *Brassica* (Guo et al., 2017). Their *Arabidopsis* orthologs were retrieved (https://www.arabidopsis.org/) for a total of 621 sequences. PSGs were also identified in poplar (Lin et al., 2018). The *Populus trichocarpa* sequences were retrieved from PopGenIE.org. Sequences of the 2,047 conifer genes under positive selection were then compared at the protein level to poplar and *Arabidopsis* proteins. Overall, pairs of homologous sequences between these dicots and conifers were identified following a blastp search (E-value<1E-30). When one dicot gene sequence was found homologous to several conifer gene sequences, or when one conifer gene sequence was homologous to several dicot gene sequences, only the best match was selected.

## Results

### SNP diversity

This study enabled the identification of nearly 1.5 million of SNPs across the transcriptomes of seven conifers (*Picea glauca* and *Picea mariana*, *Pinus strobus* and *Pinus banksiana*, *Larix laricina*, *Abies balsamea*, *Thuja occidentalis*). However, we retained the ~867K SNPs with highest quality (see Methods S5) to conduct the subsequent analyses. Among them, ~398K high-quality SNPs were located in coding sequences representing almost ~97K Open Reading Frames (equivalent to ~16K transcripts per species, on average) (**Table 1**). All species considered, ~82% of the transcripts and ~70% of the ORFs carried SNPs. In such transcriptome sequencing endeavor, it appeared important to minimize the effects caused by sequencing depth before undertaking any analysis of SNP diversity. We carefully adjusted the SNP diversity by both sequence length and sequencing depth before proceeding to data analysis and comparison across genes and across species (see Methods). After these adjustments, SNP diversity was estimated for each species and it was found significantly heterogeneous among the seven conifer taxa. A Kruskal-Wallis test revealed significant differences in rates of synonymous, nonsynonymous and total SNPs across the seven species (**Supplementary Table S2**). Based on overall SNP diversity, three groups were delineated based on the results of Kolmogorov-Smirnov and Cramer-von Mises tests (**Figure 1**; **Table 1**; **Supplementary Methods S7**). The group of species with the highest level of overall SNP diversity included the two *Picea* species, the group with the lowest diversity included *Pinus strobus* and *Thuja occidentalis*, while the three remaining species, *Abies balsamea, Larix laricina*, and *Pinus banksiana*, had intermediate overall SNP diversity (**Table 1**; **Supplementary Table S1**).

**Table 1:** Metrics about high-quality SNPs for the seven conifer transcriptome datasets, including adjusted metrics for variations in sequence length and read coverage.

Overall SNP diversity group	Species	Raw number of transcripts	Raw number of SNPs	Adjusted number of SNPs^1^	Number of transcripts with SNP(s) after adjustment^1^	Average number of SNPs per transcript after adjustment^1^	Proportion of transcripts with SNPs after adjustment^1^
Highest	*Picea glauca*	18,060	105,778	100,128	16,581	6.0	91.8%
Highest	*Picea mariana*	20,534	114,771	108,019	18,558	5.8	90.4%
Intermediate	*Pinus banksiana*	19,510	90,985	87,011	16,441	5.3	84.3%
Intermediate	*Abies balsamea*	19,487	91,311	86,564	16,521	5.2	84.8%
Intermediate	*Larix laricina*	20,950	94,359	89,877	16,935	5.3	80.8%
Lowest	*Pinus strobus*	21,795	71,576	69,867	15,412	4.5	70.7%
Lowest	*Thuja occidentalis*	19,543	64,872	62,749	13,834	4.5	70.8%

^1^After adjustment for sequence length and depth (see Methods).

In column 1, species were grouped according to their level of intraspecific molecular genetic diversity, based on statistical tests performed on adjusted SNP diversity data (Methods S7).

### Detection of genes with high SNP A/S ratios and relationship with overall SNP diversity

**Figure 2:**
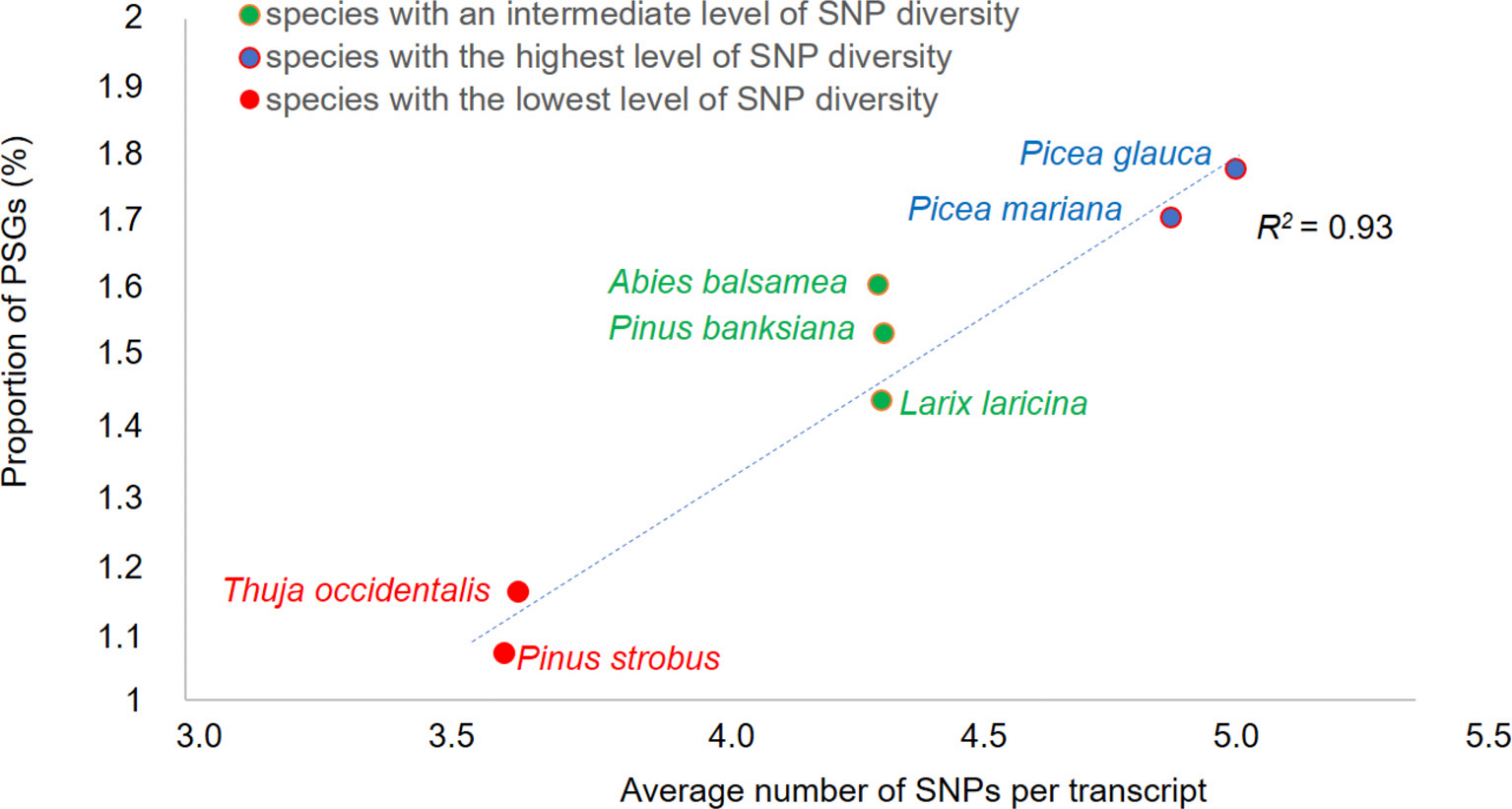
Relationship between overall SNP diversity and molecular adaptive variation. In each species, the proportion of genes under positive selection (PSGs) was calculated relatively to the total number of ORFs in the species considered. The number of SNPs was adjusted for sequence length and depth.

Synonymous and nonsynonymous sites were identified and rates of synonymous (S) and nonsynonymous SNPs (A) were estimated to calculate the gene SNP A/S ratio (see Methods). A ratio above 1 is indicative of positive or balancing selection related to adaptive evolution (Kimura, 1983; Fay et al., 2001). Ratios above 1 were found in around 19% and 25% of genes depending on the species (**Supplementary Table S8.2**). Moreover, an excess of nonsynonymous SNPs was significant in 2,047 genes (around 2% of genes within each species) (**Supplementary Table S8.2**) that were considered as genes under putative positive selection (PSGs, Positively Selected Genes) for subsequent analyses. In PSGs, the mean A/S values were in the range of 2.70-2.89 (**Supplementary Table S8.2**). The proportion of PSGs was highly correlated with the level of overall SNP diversity detected within each species (*R^2^ =* 0.93; p-value < 0.01; **Figure 2**). It should however be noted that while high A/S ratios are indicative of positive selection, further investigation would be required to validate the role of positive selection.

### Annotations of positively selected genes in conifers

Out of the 2,047 putative conifer PSGs, 73.5% (1,504 genes) had a significant match (blastp E-value <1E-15) with a SwissProt-Uniprot protein, a proportion consistent with other studies in conifers (Hart et al., 2020). Moreover, 932 genes (45.5%) had a match with 434 PFAM families (match E-value <1E-15). These annotated genes had a wide variety of functions (**Supplementary Figure S1**). Among GO terms, 1,219 Biological Processes (BPs) were assigned to these 2,047 genes. The most represented processes were directly related to signal transduction, responses to biotic stresses, as well as various related processes (**Supplementary Figure S2**). For instance, six terms describing plant responses to pathogens were found 576 times. Unsurprisingly, signal transduction, which is a common denominator of cell response to a stimulus, was the most predominant term (10.3% of the genes), along with defense response (7.7% of the genes) (**Supplementary Figures S1**-**S2**). The nicotinamide adenine dinucleotide (NAD) catabolic process was also highly represented (4.3% of the genes), which is consistent with the central role of NAD in plant defense responses (Pétriacq et al., 2013).

### Comparison of sequences, functions and processes of PSGs across conifer species

**Figure 3:**
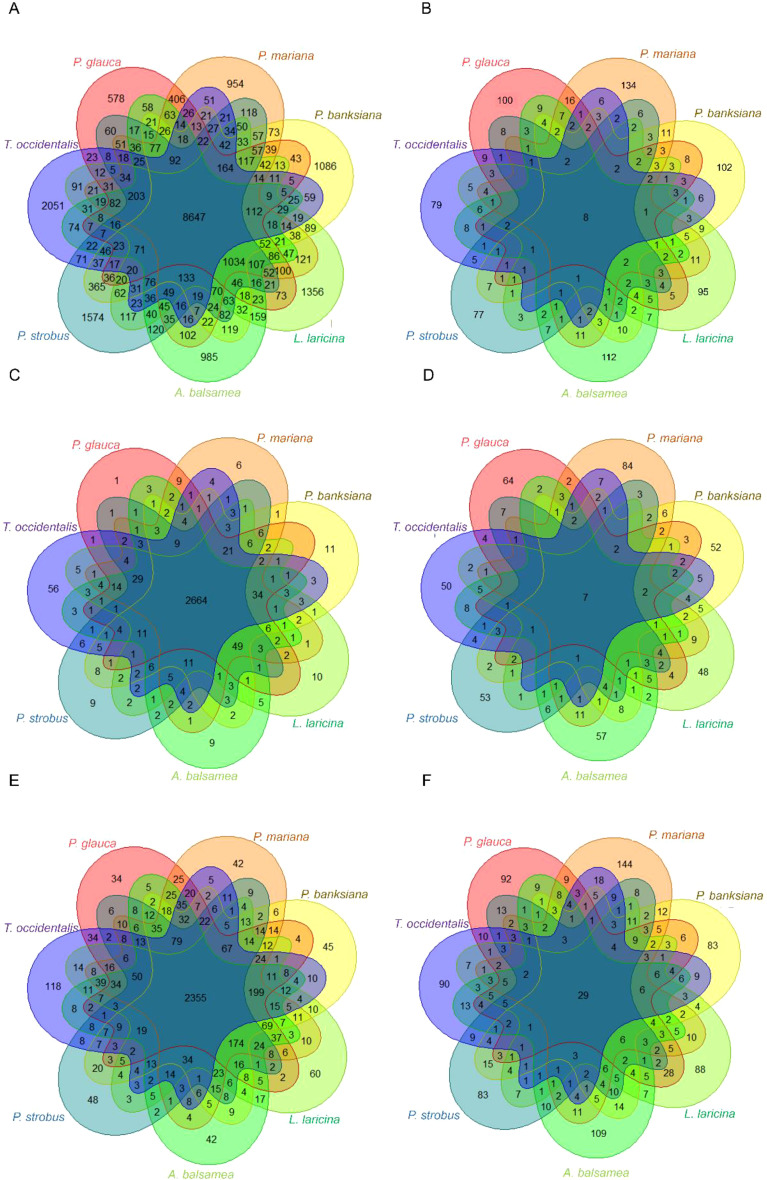
Overlap among species of orthogroups **(A, B)**, PFAM families **(C, D)** and Gene Ontology Biological Processes **(E, F)**. **(A, B)** Orthogroups were identified by clustering the complete dataset of 139k gene sequences of the seven conifer species (*Picea glauca*, *Picea mariana*, *Pinus banksiana*, *Abies balsamea*, *Larix laricina*, *Pinus strobus*, *Thuja occidentalis*) **(A)**, and by clustering the 2,047 sequences identified as positively selected genes **(B)**. The number reported in each intersection corresponds to the number of positively selected orthogroups shared by species, while the number reported in each species-specific zone corresponds to the number of singleton positively selected sequences and species-specific positively selected orthogroups. **(C, D)** Protein families were identified based on similarities against the PFAM database across the overall transcript datasets **(C)** and across positively selected genes **(D)**. **(E, F)** Biological processes GO terms across the overall sequence dataset **(E)** and across positively selected genes **(F)**.

The complete dataset of gene sequences was successfully clustered into orthogroups, demonstrating both a high clustering capability at the intraspecific level (with few unassigned genes) and at the interspecific level (with few species-specific orthogroups) (**Supplementary Table S3**). Altogether, 94.7% of the gene sequences were assigned to an orthogroup and the remainder were orphans, as previously observed in conifers (Gagalova et al., 2022). Out of the 16,982 orthogroups delimited in total, 8,647 contained gene sequences from the seven species, and 1,034 others contained gene sequences from all species except the more phylogenetically distant Cupresseae taxon *Thuja occidentalis* (**Figure 3A**). The number of species-specific genes in our dataset (hereafter referred to as ‘species-specific genes’ to simplify terminology) was low in Pinaceae taxa (between 3% and 9%) and higher (19%) in*Thuja occidentalis*, which was expected given that this taxon belongs to the more divergent Cupressaceae family (**Figure 3A**). These species-specific gene sequences could either not be assigned to any orthogroup, or represented species-specific orthogroups in our dataset (**Supplementary Table S3**). In contrast to the trend observed in the complete dataset, orthogroups derived from PSGs in each species showed a much lower overlap among species (**Figure 3B**). The vast majority of them were species-specific (total of 699; 68.7%), with only eight orthogroups shared across all seven species (**Figure 3B**; **Table 2**). No particular trend between gene convergence and phylogenetic relatedness among species was detected. For instance, shared PSGs were not more predominant within genera than among genera (**Supplementary Table S4**).

**Table 2:** Percentage of shared or species-specific genes and annotations among the complete dataset (all ORFs) and among the 2,047 positively selected genes.

Elements	Shared elements		Species-specific elements	
	complete dataset	2,047 positively selected genes	complete dataset	2,047 positively selected genes
Orthogroups	64.9%	31.3%	35.1%	68.7%
Protein families	96.8%	31.2%	3.2%	68.8%
Gene ontology terms (BP)	91.4%	59.1%	8.6%	40.9%

Sequence clustering was performed using orthofinder (Emms and Kelly, 2019) and orthogroups (OG) overlap among species (i.e. shared by two or more species) was assessed. Protein families were determined based on matches with domains or families from the PFAM database (E-value<E-15). Gene Ontology (GO) terms assigned to the Uniprot protein matching the conifer gene sequence with the lowest E-value below E-15 were used to illustrate the overlap in Biological Processes (BP) implicated in positively selected genes among species.

Similar trends were observed at the gene family level. In the overall dataset, only 102 PFAM accessions (3.2%) were species-specific, indicating that protein families or domains were predominantly shared among the seven conifer species (**Figure 3C**). In contrast, in PSGs, a majority of PFAM accessions (407 accessions, 68.8%) were species-specific (**Figure 3D**). Nevertheless, the proportion of shared families among species increased as compared to that for orthogroups (**Table 2**; **Supplementary Figure S3**).

Among GO terms, 4,504 BPs and 814 BPs were associated with the overall gene sequence dataset and 814 BPs for the dataset of PSGs (**Figure 3E**; **Supplementary Figure S4**). Species-specific BPs were few in the overall sequence dataset (total of 389, 8.6%) but relatively more abundant (333, 40.9%) in PSGs, although at a much lower rate than that observed for orthogroups or PFAM families. In spite of more convergence observed at the level of BPs, these results are indicative of a high level of functional diversity in PSGs (**Figure 3F**; **Table 2**).

The eight orthogroups of PSGs shared by all conifer species (**Figure 3B**) were homologous to sequences of known functions except one. They included three disease resistance genes, a member of pentatricopeptide repeat family, a C-type lectin receptor-like tyrosine-protein kinase, a cytochrome P450 and an ABC transporter family member.

The shared PFAM accessions included the NB-ARC domain (PF00931), the protein kinase domain (PF00069) and the TIR domain (PF01582), which are frequently found in combination in proteins involved in defense responses, as well as the cytochrome P450 family (PF00067), and UDP-glucoronosyl and UDP-glucosyl transferase family (PF00201) (**Figure 3D**; **Supplementary Figure S3**). The most abundantly represented and shared families by at least two species are detailed in **Supplementary Figure S3**.

**Figure 4:**
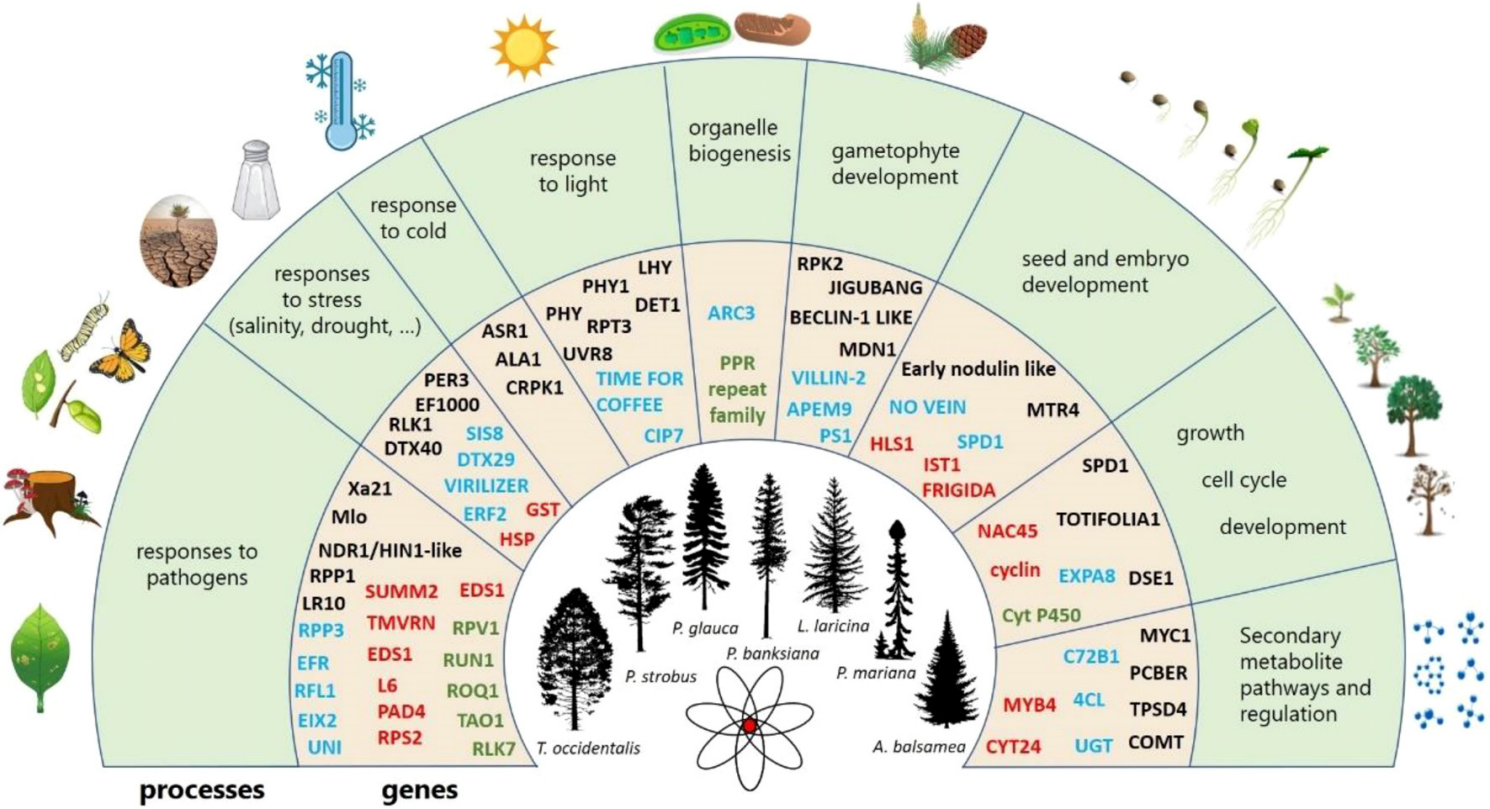
Summary of the conserved biological processes across the 2,047 positively selected genes in conifers. The outer circle shows the biological processes found with some degree of conservation across the seven species, the inner circle shows examples of genes in each functional category. Genes in black were found in a single conifer species, those in blue were found in two species, those in red were found in three to six species and green font indicates genes found in all species. Source of illustrations: https://tidcf.nrcan.gc.ca/; https://www.vecteezy.com/; https://www.freepik.com/; https://pixabay.com/.

Regarding the gene ontologies of PSGs, 29 BPs (2.38% of the total number of BPs observed in PSGs) were shared by all conifer species (**Figure 3F**) and were categorized into eight groups (**Supplementary Figure S4**). Eleven BPs were related to responses to biotic (like response to fungi, hypersensitive response) or abiotic stresses (like oxidative stress, cold) (**Supplementary Figure S4**). Four terms were related to protein modification (like protein ubiquitination) and three others were related to growth and development (like pollen development). Main shared BPs also encompassed other mechanism important for plant life and survival such as seed development and germination or response to light (**Figure 4** and **Supplementary Figure S5**).

**Figure 5:**
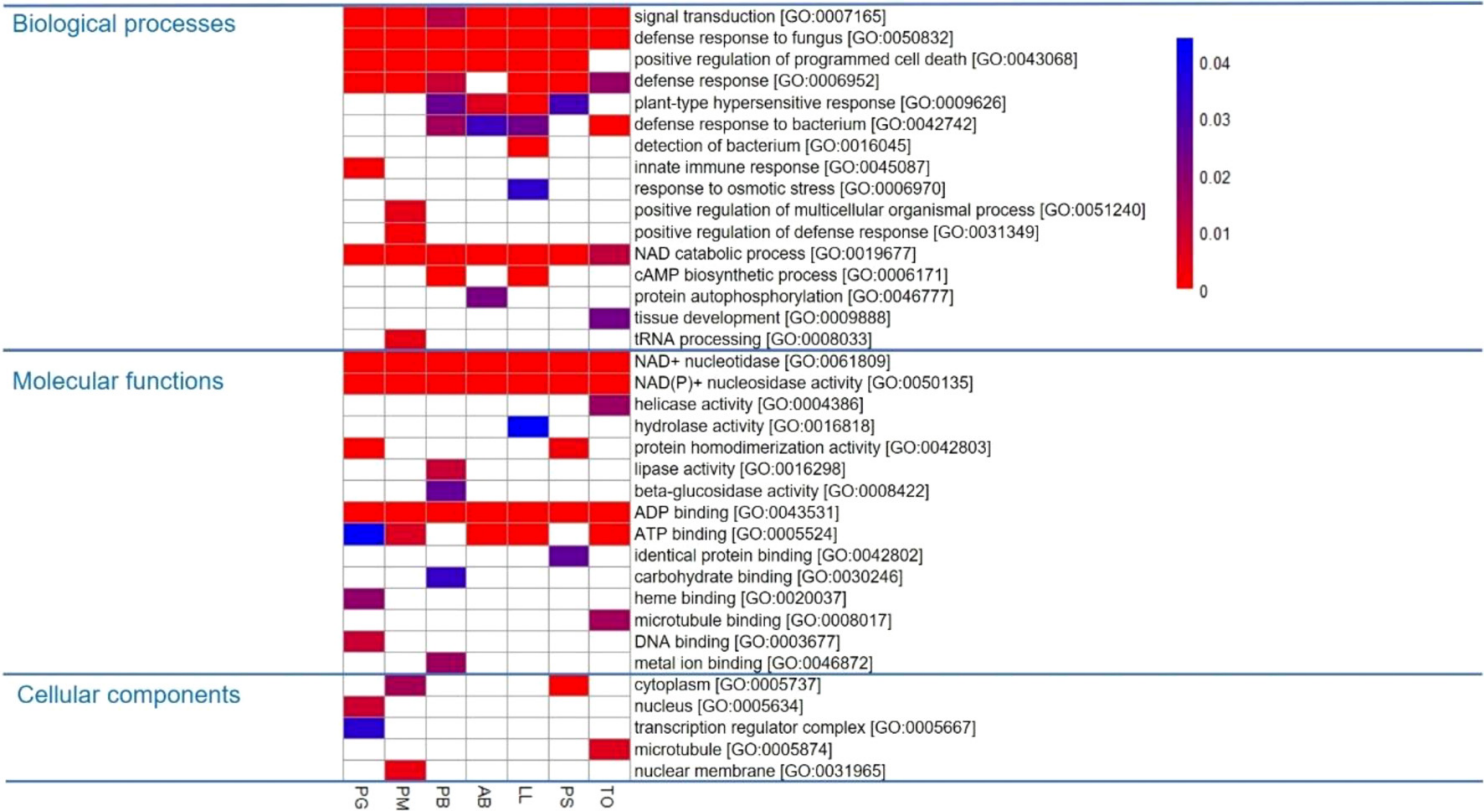
Gene Ontology terms enriched in the 2,047 positively selected genes in the seven conifer species analyzed, compared with the overall gene set for these species. (PG: *Picea glauca*, PM: *Picea mariana*, PB: *Pinus banksiana*, AB: *Abies balsamea*, LL: *Larix laricina*, PS: *Pinus strobus*, TO: *Thuja occidentalis*). The heatmap is based on the p-values of the enrichment tests (Fisher tests) and the color scale illustrates statistical significance. White cells represent non-significant tests at a threshold of 0.005.

PSGs were enriched in several GO classes, including 16 BPs, 15 molecular functions and 5 cellular components (**Figure 5**). Among them, five terms were enriched in the seven conifer species and are involved in defense against pathogens. Half of the enriched BPs were related to stress responses (**Figure 4**). The enriched molecular functions were involved in several enzymatic activities or in binding (**Figure 5**). Noticeably, terms involving nucleotidases, ADP or ATP binding were enriched in almost all seven species (**Figure 5**).

### Abundance of conifer PSGs in defense mechanisms

Several gene families representing PSGs were involved in disease resistance. Among resistance genes, there were 72 genes homologous to *run1* and 41 homologous to *rpv1* conferring resistance to mildew (**Supplementary Table S5**). Several gene families were involved in resistance against *Pseudomonas syringae* (*rps2*, *rps5*, *rfl1*, *tao1*, *rpp3*) or against viruses (*eds1*, *tmvrn*) (**Supplementary Table S5**, **Figure 4**). Among families involving PSGs shared by all species, the C-Lectin (CLEC) and the DRL28 protein are also involved in disease resistance. CLEC proteins have a diverse range of functions including cell-cell adhesion, immune response to pathogens and apoptosis.

Several genes belonged to the chitin pathway involved in fungal wall degradation (**Supplementary Table S6**). They included several chitinases, an homologue of the LYK5 chitin receptor (Cao et al., 2014), and an homologue of CERK1 (Chitin Elicitor Receptor Kinase 1) required as a cell surface receptor for chitin elicitor signaling leading to innate immunity in response to biotic stresses (Hu et al., 2021).

Also, several PSGs were found among gene families implicated in the secondary metabolite pathways (**Figure 4**, **Supplementary Table S7**). They included proteins with roles in wood formation and defense response against insects such as the cytochrome P450 76T24 involved in the monoterpenoid synthetic pathway (Miettinen et al., 2014), the abietadienol/abietadienal oxidase (Ro et al., 2005), and the delta-selinene synthase (Steele et al., 1998).

### Conifer PSGs homologous to Brassica or poplar PSGs

Conifer PSGs had 54 homologs also positively selected in both poplar and *Brassica*. This set of 54 PSGs matched a total of 17 distinct *Arabidopsis* genes including five transcription factors, three glucosyltransferases, two peroxidases and a range of other gene families (**Table 3**).

**Table 3:** Description of 17 *Arabidopsis* genes whose homologs are positively selected genes in at least one conifer species and in both poplar (Lin et al., 2018) and *Brassica* (Guo et al., 2017).

Locus Identifier	Gene names	Protein family (known function)
AT2G40270		Protein kinase family protein
AT4G29270		HAD superfamily, subfamily IIIB acid phosphatase
AT5G03610	GGL25	GDSL-motif esterase/acyltransferase/lipase
AT3G14330	CHLOROPLAST RNA EDITING FACTOR 3 (CREF3)	pentatricopeptide repeat protein (involved in chloroplast mRNA editing)
			AT2G36800	UDP-GLUCOSYL TRANSFERASE 73C5 (UGT73C5)	DON-Glucosyltransferase (presumably involved in the homeostasis of those steroid hormones)
DON-GLUCOSYLTRANSFERASE 1 (DOGT1)					
AT1G78380	GLUTATHIONE S-TRANSFERASE TAU 19 (GSTU19)	glutathione transferase from the Tau GST gene family (Expression is induced by drought stress, oxidative stress, and high doses of auxin and cytokinin)
	GLUTATHIONE TRANSFERASE 8 (GST8)	
AT5G15150	HOMEOBOX 3 (ATHB-3)	homeobox-containing gene
	HOMEOBOX ARABIDOPSIS THALIANA 7 (HAT7)	
AT5G16600	MYB DOMAIN PROTEIN 43 (ATMYB43)	MYB family of transcription factors Encodes a transcriptional regulator that directly activates lignin biosynthesis genes and phenylalanine biosynthesis genes during secondary wall formation.
AT5G10280	MYB DOMAIN PROTEIN 92 (ATMYB92) (ATMYB64)	MYB family of transcription factors
AT5G35550	MYB DOMAIN PROTEIN 123 (AtMYB123) TRANSPARENT TESTA 2 (TT2)	MYB family of transcription factors (acts as a key determinant in the proanthocyanidin accumulation of developing seed)
AT3G10480	NAC DOMAIN CONTAINING PROTEIN 50 (NAC50)	Encodes a NAC transcription factor that physically associates with the histone H3K4 demethylase JMJ14 and through that association is involved in transcriptional repression and flowering time control.
			AT5G05340	PEROXIDASE 52 (PRX52)	peroxidases (involved in lignin biosynthesis)
AT3G49120	PEROXIDASE 34 (PRX34)	peroxidases (involved in cell elongation. Expression activated by light. May play a role in generating H2O2 during defense response.)
AT1G17020	SENESCENCE-RELATED GENE 1 (ATSRG1)	Fe(II)/ascorbate oxidase gene family (senescence-related gene)
AT3G25420	SERINE CARBOXYPEPTIDASE-LIKE 21 (scpl21)	serine carboxypeptidase-like
AT2G36750	UDP-GLUCOSYL TRANSFERASE 73C1 (UGT73C1)	UDP-glucosyl transferase
AT3G46670	UDP-GLUCOSYL TRANSFERASE 76E11 (UGT76E11)	UDP-glucosyl transferase

**Figure 6:**
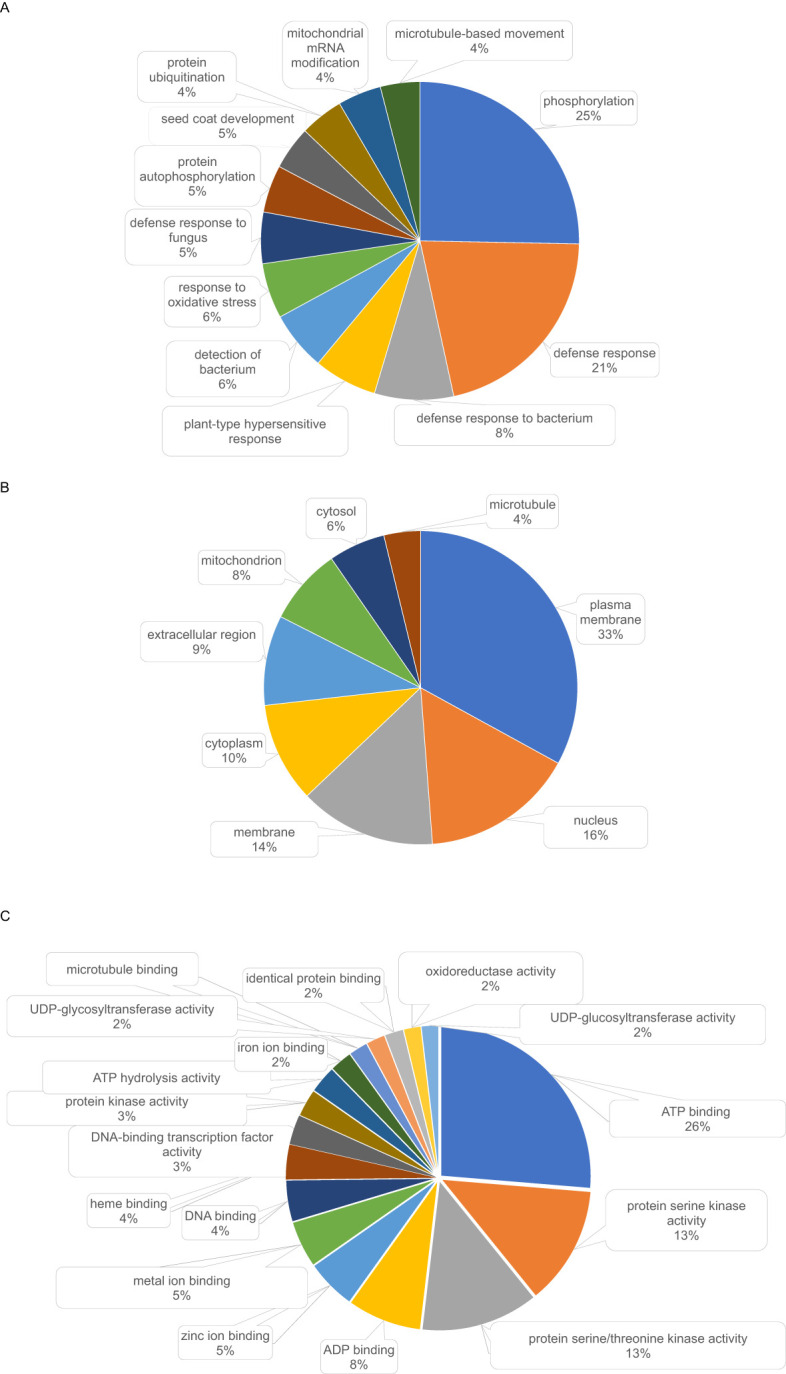
Most represented GO terms (found 10 times or more) in annotations of the 384 positively selected conifer genes found homologous (blastp E-value<1E-30) to positively selected genes in poplar (Lin et al., 2018) or *Brassica* (Guo et al., 2017). **(A)** biological processes, **(B)** cellular components, **(C)** molecular functions.

In addition, 384 conifer PSGs were homologous to PSGs in either poplar or *Brassica*. Their annotations were diverse (**Figure 6**) and related to a variety of BPs (**Supplementary Table S8**). The most represented BPs in this group were related to stress responses (164 genes) and metabolism (127 genes) (**Figure 6**). Several genes were homologous to disease resistance genes including several members of the *rps* family and homologs to *summ2* (SUPPRESSOR OF MKK1 MKK2 2) conferring resistance against *Pseudomonas syringae*, as well as *lrks4* and *lrks7* (lectin-domain containing receptor kinase involved in resistance response to the pathogenic oomycetes *Phytophthora*) (**Supplementary Table S5**).

## Discussion

### Contrasting levels of molecular genetic diversity among conifers

Several lines of evidence indicated that levels of total molecular genetic diversity differed substantially among the seven conifers (**Table 1**; **Supplementary Methods S7**; **Supplementary Table S2**). *Pinus strobus* and *Thuja occidentalis* had the lowest overall SNP diversity in their transcriptome, the two spruces were the most diverse, and the three remaining species were intermediate (**Figure 1**). Several factors related to the experimental design may result in an underestimation of the intraspecific diversity estimates reported herein. For instance, rare alleles may be missed out due to their low frequencies in sequencing pools. Moreover, diversity estimates rely only on gene expressed in the embryo, which nonetheless represents roughly 60% of the transcriptome (Rigault et al., 2011). However, our comparative analyses should not be biased by these limitations, as they would affect all species equally. Interpreting transcriptome-wide genetic diversity patterns is not straightforward because intraspecific variation results from the complex interplay between mutation rate, effective population size (long-term *N*e, which depends itself on historical and demographic factors), and linked selection (the molecular genetic diversity-reducing effect of selective sweeps on neutral loci in linkage with loci under natural selection) (Ellegren and Galtier, 2016).

The seven conifer species analyzed here are not expected to have significantly different mutation rates, as they are all long-lived woody perennials (Petit and Hampe, 2006; Sung et al., 2012) and considering that our genetic diversity estimates derive from transcriptome-wide SNP data rather than a limited number of genes. However, in relation to the neutral theory of evolution which assumes that much of the standing genetic variation derives from neutral or nearly neutral mutations (e.g. Kimura, 1983; Ohta, 1992), part of the observed interspecific differences in molecular genetic diversity likely relates to historical effective population sizes (Bousquet et al., 1992). Indeed, the minimum historical population size (*N*e) of *Picea glauca* and *Picea mariana*, the most diverse species group in this study, was estimated at ~100,000 or more individuals (Bouillé and Bousquet, 2005; Chen et al., 2010), while that of *Pinus strobus*, which belongs to the low diversity group, has been estimated to be an order of magnitude lower (5000 to 10,000 individuals; Zinck and Rajora, 2016). Likewise, *Thuja occidentalis*, the less genetically diverse species studied herein, also harbors a low *N*_e_ (Pandey and Rajora, 2012). There was also an apparent relation between the level of intraspecific SNP diversity and the geographical extent of the species range (**Figure 1**), though the study of more species would be needed to confirm this trend. For instance, the two most diverse species have wide transcontinental distributions, while the two less diverse species are the most geographically restricted. As the number of glacial lineages of the Pleistocene era is usually positively related with range size in North American tree taxa (Jaramillo-Correa et al., 2009), widely-distributed species are likely to have retained larger historical population size and standing genetic variation than species with currently more restricted natural ranges.

Since our molecular genetic diversity estimates originate from transcriptomic data, it is very likely that selection also played a role in shaping molecular genetic diversity because the transcriptome mostly encodes functional information. Indeed, natural selection can constrain intraspecific molecular genetic diversity through selective sweeps (Smith and Haigh, 1974; Hermisson and Pennings, 2005). Although selective sweeps are presumably uncommon in plants (Wright and Gaut, 2005; Grossman et al., 2010) including in conifers (Palme et al., 2008; Pavy et al., 2012b; Eckert et al., 2013), evidence for selective sweeps has been reported previously in some conifers (Eckert et al., 2009; Namroud et al., 2010; Wang et al., 2020; De La Torre et al., 2021; Gagalova et al., 2022). The pool sequencing data obtained for each species in our study does not allow to test directly for the existence of such sweeps. However, it is unlikely that selective sweeps are a major determinant of transcriptome-wide genetic diversity in conifers because, on average, linkage disequilibrium decays rapidly within gene limits in conifer natural populations (e.g. Pavy et al., 2012b; De La Torre et al., 2017), therefore restricting the possible loss of neutral diversity surrounding selected loci. Hence, although both *N*_e_ and linked selection may have contributed to shaping molecular genetic diversity at the intraspecific level, the former is more likely to have been the main driver of differences in overall SNP diversity observed herein among the transcriptomes of the studied species.

### Relationships between overall SNP diversity and molecular variation of adaptive nature

The positive relationship observed between the overall SNP diversity of the transcriptome of each species and their proportions of PSGs (**Figure 2**) suggests that standing genetic variation can constrain variation of more adaptive nature. It is well established that the fixation probability of favorable alleles in a finite population increases along with the effective population size by reducing the strength of genetic drift and therefore limiting the loss of beneficial alleles as well as the fixation of deleterious ones (Charlesworth, 2009). Consistently, our results show that species of presumably larger historical population sizes carry the most adaptive molecular variation. This is also in agreement with more common local adaptation in large plant populations than in small ones (Leimu and Fischer, 2008), which has been reported for several species investigated herein, namely white spruce (Namroud et al., 2008; Hornoy et al., 2015; Depardieu et al., 2021), black spruce (Prunier et al., 2011; 2012), jack pine (Cullingham et al., 2014), and eastern white pine (Nadeau et al., 2016). Because environmental adaptation is highly polygenic in conifers and involves heterogeneous gene responses (e.g. Hornoy et al., 2015; Yeaman et al., 2016; Depardieu et al., 2021), high standing genetic variation associated with large historical population size likely improves species adaptative potential by increasing the number of possible genetic trajectories to achieve adaptation. Hence, such flexibility may allow species to cope with a wider range of environmental conditions (i.e. gain the ability to colonize larger natural range and/or increase their ecological amplitude) and selective pressures (i.e. biotic and abiotic pressures encountered across their range).

### Extent of molecular and functional convergence among conifer adaptive genes

Despite the high overlap among gene sequences of the seven conifer species (**Figure 3A**), we only found limited molecular convergence among their genes under positive selection (**Figure 3B**). Convergence appeared also limited at the protein family level (**Figure 3D**), a result consistent with the pattern of species-specific expansion of large paralogous gene families (reviewed by De La Torre et al., 2020) and high functional redundancy in conifers (Guillet-Claude et al., 2004; Bedon et al., 2010; Stival Sena et al., 2018; Van Ghelder et al., 2019). The extent of molecular genetic convergence among taxa would also be expected to increase with their phylogenetic proximity, as a result of shared ancestry (Losos, 2011; Storz, 2016). However, no pattern related to phylogenetic relatedness among taxa was evident, with no sign of increased convergence among the two pairs of congeneric taxa that would have diverged the most recently (divergence between *Picea glauca* and *Picea mariana* ~10 Mya (Bouillé and Bousquet, 2005); divergence between *Pinus strobus* and *Pinus banksiana* ~85 Mya (Leslie et al., 2018) (**Supplementary Table S4**). Similarly, convergence was only marginally higher among the Pinaceae taxa that between the Pinaceae taxa and the Cupressaceae taxon despite their more recent divergence (Leslie et al., 2018) (**Supplementary Table S4**). These observations suggest that each species followed a largely distinct adaptive path, and that adaptive convergence at the molecular genetic level appears to be limited in such reproductively isolated and phylogenetically distant conifers.

There is also strong evidence that the marked difference in sets of PSGs among species would be primarily driven by natural selection, rather than by stochastic processes such as mutation or genetic drift (e.g. Losos, 2011; Mosca et al., 2012; Storz, 2016). The low levels of convergence observed among the species gene sets under positive selection either indicate that gene functional redundancy would allow species to cope with similar selective pressures using alternative genes, and/or that species experienced heterogeneous selective pressures throughout their historical and extent natural ranges. Our data support the first hypothesis, as functional convergence among biological processes associated with genes under positive selection was quite higher that molecular convergence among these genes (**Figure 3**). This partly decoupled pattern indicates that the studied species would have had sufficient metabolic and gene network flexibility to evolve alternative responses to the various selective pressures they faced under temperate and boreal climate regimes. It is also consistent with gene family expansions in conifers, implying some redundancy in gene functions (Guillet-Claude et al., 2004; Bedon et al., 2010; Pavy et al., 2012a; Stival Sena et al., 2018; Van Ghelder et al., 2019). This redundancy at the functional level may have had significant evolutionary implications for the persistence of these northern conifer species during millions of years, in the face of geological climate instability and in spite of demographic fluctuations. For instance, with the multiple glaciation cycles of the Pleistocene era in northeastern North America, signatures of demographic fluctuations such as bottleneck effects or founder events have been detected in various conifer species from this region, which would have implied more or less important losses of overall genetic diversity (Perron et al., 2000; Gamache et al., 2003; Jaramillo-Correa and Bousquet, 2003; Godbout et al., 2010; Namroud et al., 2010).

In addition to the gene redundancy hypothesis, it is possible that the low convergence observed among conifer PSG sets also reflects that the species have coped with specific long-term selective pressures throughout their large natural ranges. Some species such as the two *Picea* spp. have large ecological amplitude and a transcontinental range across which they may encounter a variety of selective pressures related to biotic and abiotic stresses (Nienstaedt and Zasada, 1990; Viereck and Johnston, 1990). Also, *Picea glauca* would prefer mesic sites (Hornoy et al., 2015) while *Picea mariana* could adapt to a larger variety of site conditions (Lo et al., 2024). Others have more distinct preferred habitats, such as wetter sites for *Larix laricina* (Cheliak et al., 1988), dryer sites for *Pinus banksiana* (Rudolph and Laidly, 1990), or both for the more extremophile *Thuja occidentalis* (Matthes-Sears and Larson, 1991), which can trigger specific adaptive responses and explain the finite extent of adaptive convergence in gene sets under positive selection among the conifer species studied.

Hence, taken together, our results suggest that the adaptive trajectories of these conifer species were likely shaped by the interplay of gene redundancy and heterogeneous selection landscape, and that these two drivers likely contributed to the low convergence observed in terms of gene sets accumulating nonsynonymous SNPs, but higher convergence at the functional level.

This pattern aligns well with other reports of low estimates of molecular genetic convergence among conifers. A study of adaptive traits in four alpine conifers from the *Pinus*, *Abies* and *Larix* genera identified only seven climate-associated genes shared by two or more species out of several hundreds of sequences analyzed (Mosca et al., 2012). Another study uncovered only 47 convergent genes (representing between 10% and 18% of all genes putatively under selection) involved in local adaptation in a spruce and a pine taxon that diverged ~150 Mya (Yeaman et al., 2016). Also, a transcriptome-wide survey of genetic variation in the two quite closely-related but ecologically contrasted *Picea glauca* and the coastal *Picea sitchensis* from the Pacific Northwest, revealed only 15 shared genes out of hundreds of genes showing selection footprints (Gagalova et al., 2022). Similar modest molecular genetic convergence was also reported in Angiosperms such as in *Arabidopsis* (Guggisberg et al., 2018; Preite et al., 2019), or among taxa from the Brassicaceae family (Rellstab et al., 2020).

### Genes under positive selection in conifers and overlap with angiosperms

Functional annotations of genes under positive selection were associated with a great variety of molecular functions and biological processes (**Supplementary Figure S1**), in agreement with the polygenic nature of adaptive traits and the many empirical studies reporting a wide range of genes and functions underlying them in conifers (e.g. Prunier et al., 2011; Mosca et al., 2012; Hornoy et al., 2015; Depardieu et al., 2021).

Despite the large functional diversity observed, the core set of functions and processes shared by all conifer species analyzed revealed a clear pattern of shared adaptive evolution at the functional level. Indeed, half of the 29 shared BPs by all conifer taxa examined herein were related to environmental stress responses, and mechanisms related to defense against pathogens (responses to biotic stress, programmed cell death) were widely represented and enriched in genes under positive selection (**Figure 4**; **Figure 5**). We found many homologs of disease resistance genes with the NB-ARC domain (**Supplementary Figure S4**), several gene families involved in resistance to *Xanthomonas*, *Pseudomonas* or rusts (**Figure 4**; **Supplementary Table S5**), and several genes encoding enzymes from the secondary metabolite pathways that are known to be involved in defense mechanisms as well as wood formation (**Supplementary Table S7**). This indicates that selection pressures exerted by pathogens are likely ubiquitous in conifers and play a prominent role in their adaptation to changing environments. Similarly, several shared BPs were linked to abiotic stress response, and more specifically to water and oxygen stimuli (i.e. cellular response to hypoxia, response to oxidative stress, or response to water deprivation), suggesting that drought and flooding could also be drivers of adaptive evolution at the functional level in conifers.

Mechanisms with crucial roles in stress response such as RNA modification and regulatory mechanisms were also found in conifer genes under selection (**Figure 4**). Regarding RNA modification, we identified 59 sequences encoding pentatricopeptide repeat (PPR) proteins, among which two were also reported as convergent adaptive genes in pine and spruce taxa (**Figure 4**, **Supplementary Figure S4**; Yeaman et al., 2016). The PPR proteins have fundamental roles in organelle biogenesis and function, being involved in photosynthesis, respiration, development and environmental responses (Barkan and Small, 2014). Positive regulation of programmed cell death, a process known to be involved in response to biotic and abiotic stresses in plants (Daneva et al., 2016), was also enriched in all Pinaceae species (**Figure 5**). Transcriptional regulators included several MYB and WRKY gene sequences. Homologs of genes involved in seasonal transitions in *Arabidopsis* are likely prime targets of natural selection given that they contribute to the adaptation of plants to their environment, assuming that their functions are conserved across seed plants. Indeed, a FRIGIDA-like protein (accession FRL3_ARATH) was found among conifer genes under positive selection (**Figure 4**). In *Arabidopsis*, it regulates phase transition during shoot, flower and seed development such that FRIGIDA gene sequences are required for the winter-annual habit (Michaels et al., 2004). The identification of several homologous regulators involved in survival in Angiosperms also suggests a possible key role in conifers and makes them a relevant class of genes to target in future molecular functional studies.

We uncovered 17 PSGs shared by conifers and two Angiosperm taxa (Brassicaceae and poplar) (**Table 3**). This level of molecular convergence was higher than expected, given that Angiosperms and Gymnosperms (to which belong conifers) diverged ~350 Mya (Li et al., 2019). Disease resistance genes against pathogens are known to evolve rapidly in flowering plants (Meyers et al., 2005). Although well represented among conifer PSGs, they were not predominant among the core set of convergent genes between conifers and Angiosperms. In contrast, several genes encoding enzymes (transferases, peptidase, peroxidases), one PPR gene involved in RNA editing, and several transcription factors such as MYBs showed up in this set of genes (**Table 3**). To our knowledge, such widely spread molecular signatures of adaptation have not been reported to date and may be interpreted as a sign that adaptive convergence at the molecular level, though limited, can take place at a very broad taxonomic level. Consequently, these genes represent valuable candidates for future evolutionary studies aiming to characterize molecular and functional convergence among seed plants.

## Conclusions

Transcriptome-wide SNP diversity was assessed for seven partially sympatric and reproductively isolated conifers. We found marked variation in overall SNP diversity among species, that would reflect mainly differences in demography and historical population size. Little overlap in sets of adaptive genes under positive selection was noted among species, suggesting distinct evolutionary trajectories. In contrast, their biological functions were much convergent and largely related to stress response and regulatory mechanisms. This trend indicates high molecular plasticity in response to similar climate and natural selective pressures. Several adaptive gene homologs were shared between conifers and Angiosperms, despite their ancient divergence ~350 Mya.

## Data Availability

The datasets presented in this study can be found in online repositories. The names of the repository/repositories and accession number(s) can be found below: https://www.ebi.ac.uk/ena, ERS16017105-ERS16017139 and ERS16049778-ERS16049791; https://doi.org/10.5061/dryad.p8cz8w9w1, Dryad.
