# Supplementary material for: Contrasting levels of transcriptome-wide SNP diversity and adaptive molecular variation among conifers
Source: Front Plant Sci. 2025 Mar 6;16:1500759. doi: 10.3389/fpls.2025.1500759 (PMC11922845; doi:10.3389/fpls.2025.1500759)
Supplement: Supplementary file 1 [file DataSheet1.pdf]

Supplementary material

Supplementary experimental procedures

Methods S1

The geographic origins of pools of embryos and single megagametophytes sequenced for each of the seven conifer species are described in Table S1.1, together with the numbers of embryos and megagametophytes sampled.

Table S1.1 Geographic origins of sequenced material.

Species	NTSC[1]_lot_id	Provenance	Province[2]	Latitude	Longitude	Number of embryos in the species pool	Number of individual megagametophyte
Picea glauca	7965049	Df 110-16-5-79	AB	58.63334	-114.95000	1	1
Picea glauca	8200570	Goose Bay	NL	53.33333	-60.16667	2	1
Picea glauca	8421507	St-Gedeon	QC	45.83333	-70.65000	2	0
Picea glauca	8431739	Seed Zone 18	ON	49.50000	-80.50000	2	1
Picea glauca	8810033	Killag Mines	NS	45.00000	-62.60000	2	0
Picea glauca	9350084	Spiritwood	SK	53.75000	-107.71670	2	1
Picea glauca	9740087	Churchill	MB	58.83333	-94.11667	1	0
Picea glauca	20015157	Hudson Bay	SK	54.11666	-102.65000	2	0
Picea glauca	20022383	Lac-Cadilac	QC	48.38334	-78.13333	2	0
Picea glauca	20034032	Beaver Creek	MB	51.41667	-96.93333	2	0
Picea mariana	8233230	Chapleau Highlands	ON	47.86666	-83.16666	2	1
Picea mariana	8630180	Weagamow Indian Reserve	ON	53.00000	-91.33334	2	0
Picea mariana	8731127	Hearst	ON	50.13334	-83.83334	0	1
Picea mariana	8820074	Notre-Dame-du-Rosaire	QC	48.75000	-71.55000	2	0
Picea mariana	9020069	Lac-Deroussel	QC	50.66667	-74.00000	1	0
Picea mariana	9170004	Clear Creek	YT	63.61666	-137.51670	2	1
Picea mariana	9330173	Seed Zone 11	ON	49.00000	-92.00000	2	0
Picea mariana	9920142	Lac-Joutel	QC	49.46667	-78.13333	2	0
Picea mariana	20045962	Weyakwin	SK	59.65000	-106.08000	2	1
Pinus banksiana	6921205	Trinity Bay	QC	49.41667	-67.33334	2	1
Pinus banksiana	8021950	Briand	QC	46.90000	-76.03333	2	1
Pinus banksiana	8220072	Val-Paradis	QC	49.38334	-79.41666	1	1
Pinus banksiana	8540047	Duck Mountains	MB	51.58333	-101.00000	2	1
Pinus banksiana	8940116	Belair Forest Reserve	MB	50.55000	-96.15000	2	0
Pinus banksiana	9040116	Thompson	MB	55.76667	-97.81667	1	0
Pinus banksiana	9630232	Cyril Lake	ON	50.50000	-86.41666	1	0
Pinus banksiana	20002285	Poste-de-Mistassini	QC	50.85000	-73.10000	2	0
Pinus banksiana	20043968	Seed Zone 8	ON	52.00000	-92.50000	3	0
Pinus banksiana	20073182	Seed Zone 23	ON	47.25000	-84.50000	2	0
Abies balsamea	7020013	Lac-Rouillard	QC	48.08333	-77.83334	1	1

Abies balsamea	8410903	Bear River	NS	44.58333	-65.66666	2	1
Abies balsamea	9620271	Lac-Rerock	QC	50.01667	-69.33334	4	1
Abies balsamea	9810236	Astle	NB	46.41667	-66.46667	1	0
Abies balsamea	9810243	Mechanic Settlement	NB	45.66667	-65.16666	2	1
Abies balsamea	9810245	Marie	PE	46.40000	-62.65000	4	0
Abies balsamea	9820315	Lac-Etchemin	QC	45.33333	-70.91666	2	0
Abies balsamea	9820318	St-Joseph	QC	46.33333	-70.90000	1	0
Larix laricina	8232790	Manitouwadge	ON	49.25000	-86.00000	2	1
Larix laricina	8310132	Dromore	PE	46.26667	-62.86666	1	0
Larix laricina	8421597	Lac-Morin	QC	47.83333	-69.38333	2	1
Larix laricina	8510019	Cleveland	NS	45.66667	-61.25000	2	1
Larix laricina	9110007	Granville Beach	NS	44.75000	-65.50000	2	1
Larix laricina	9220395	Lac-Maskinonge	QC	46.33333	-73.38333	1	0
Larix laricina	9220400	Lac-Abitibi	QC	48.83333	-79.16666	2	0
Larix laricina	9230443	Foymount	ON	45.43333	-77.31667	2	0
Larix laricina	9810224	Central Blissville	NB	45.68333	-66.55000	2	0
Larix laricina	9810298	West Mabou	NS	46.08333	-61.45000	2	0
Pinus strobus	8431743	Seed Zone 37	ON	43.00000	-81.00000	2	1
Pinus strobus	9620307	Lac-Taureau	QC	46.76667	-73.90000	2	1
Pinus strobus	9710048	Caribou Depot	NB	47.58333	-66.25000	2	1
Pinus strobus	9720080	Baie-Cascouia	QC	48.45000	-71.46667	2	0
Pinus strobus	9800297	Flat Bay Brook	NL	48.30000	-58.60000	2	0
Pinus strobus	20061387	Kejimkuj+M48:P48ik National Park	NS	44.41102	-65.22330	0	1
Pinus strobus	20073172	Seed Zone 14	ON	49.00000	-89.00000	1	0
Pinus strobus	20073175	Seed Zone 35	ON	44.50000	-78.00000	2	0
Pinus strobus	20073176	Seed Zone 29	ON	45.50000	-78.00000	2	0
Pinus strobus	20113132	Seed Zone 25	ON	47.00000	-82.00000	2	0
Thuja occidentalis	20001235	Glencoe	NB	47.90000	-66.81667	1	1
Thuja occidentalis	20021168	Semiwagan	NB	46.75000	-65.60000	1	0
Thuja occidentalis	20061229	Parkers Cove	NS	44.80490	-65.55205	1	0
Thuja occidentalis	20063273	Petawawa Research Forest	ON	46.11625	-77.51006	2	0
Thuja occidentalis	20063436	Seed Zone 29	ON	45.50000	-78.00000	1	0
Thuja occidentalis	20083174	Seed Zone 36	ON	44.50000	-76.50000	2	1
Thuja occidentalis	20083176	Seed Zone 38	ON	43.00000	-82.00000	2	1
Thuja occidentalis	20111045	Kingston	NB	45.37763	-66.19737	2	1
Thuja occidentalis	20113028	Thunder Bay	ON	48.39984	-89.21833	1	0
Thuja occidentalis	20113140	Seed Zone 34	ON	44.25000	-79.00000	2	0

[1]NTSC: National Tree Seed Center of Canada

[2]AB, Alberta; MB, Manitoba; NB, New Brunswick; NL, Newfoundland; NS, Nova Scotia; ON, Ontario; PE, Prince Edward Island; QC, Quebec; SK, Saskatchewan; YT, Yukon Territory

The natural range of each studied species, as well as the geographic origin of samples genotyped are illustrated on Fig S1.1-7.

Fig S1.1 *Picea glauca*

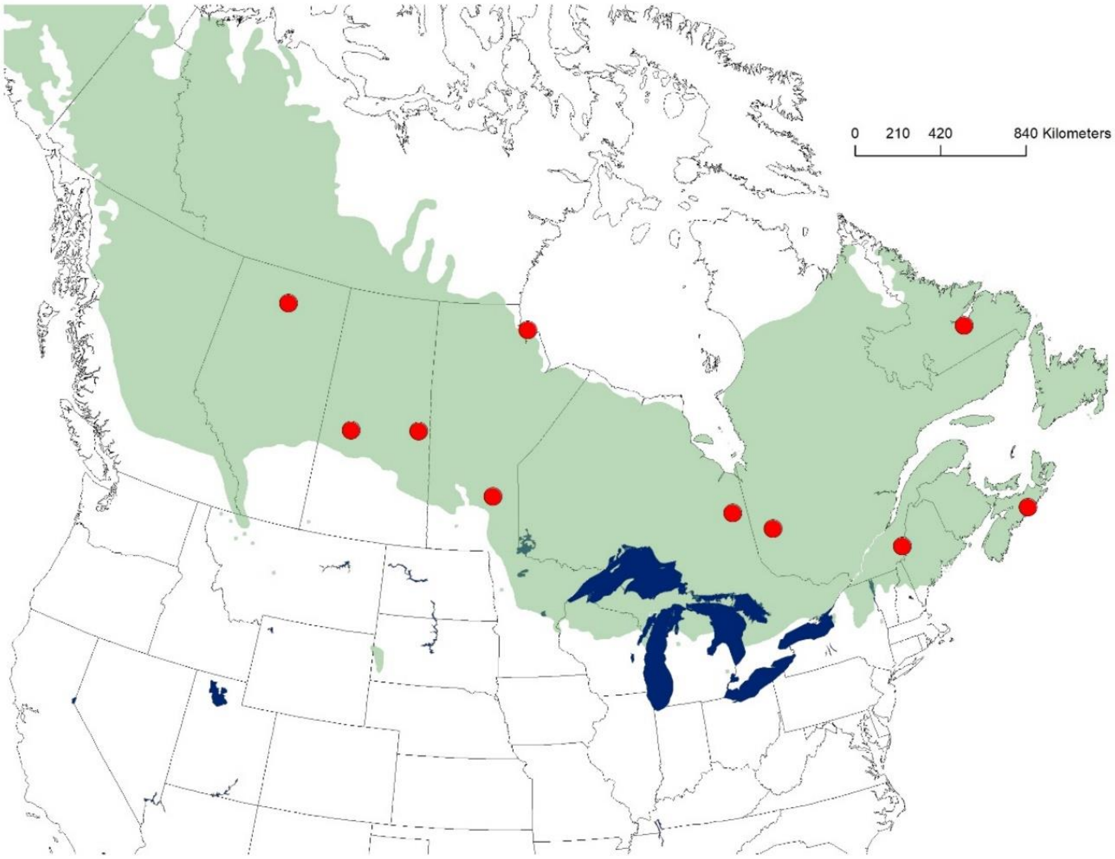

Fig S1.2 *Picea mariana*

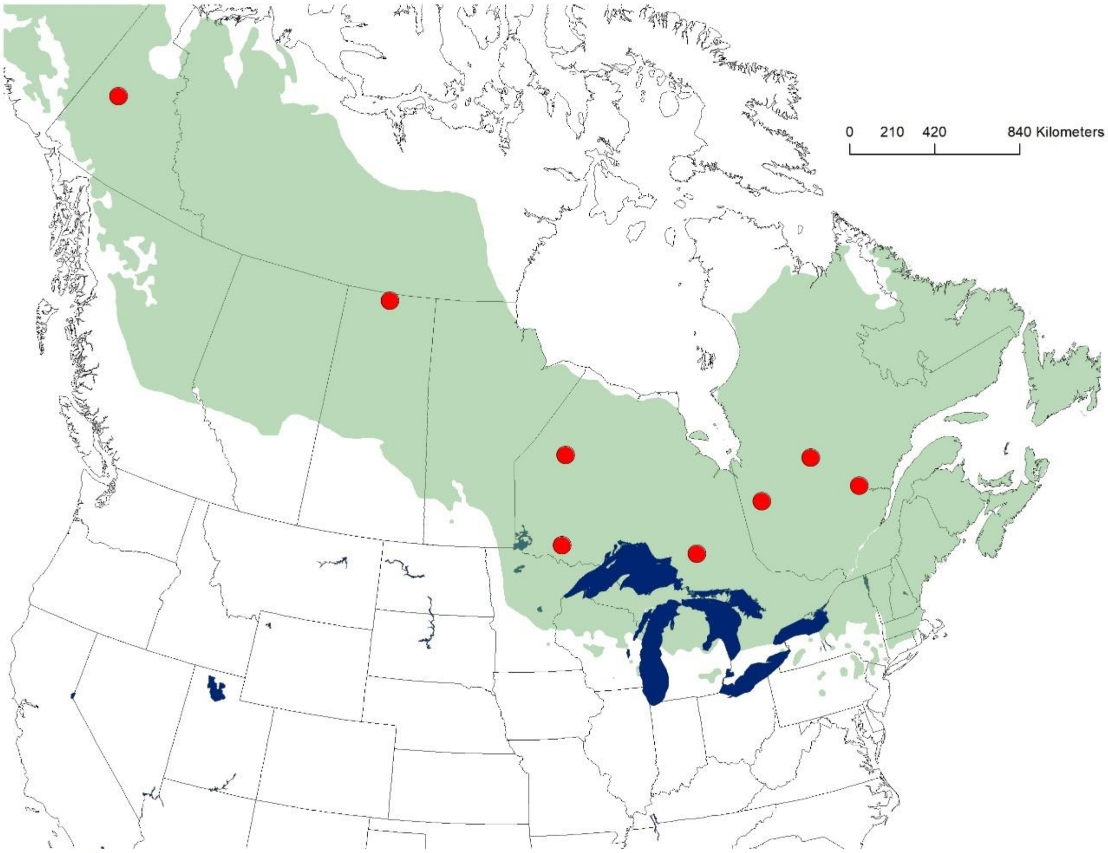

Fig S1.3 *Pinus banksiana*

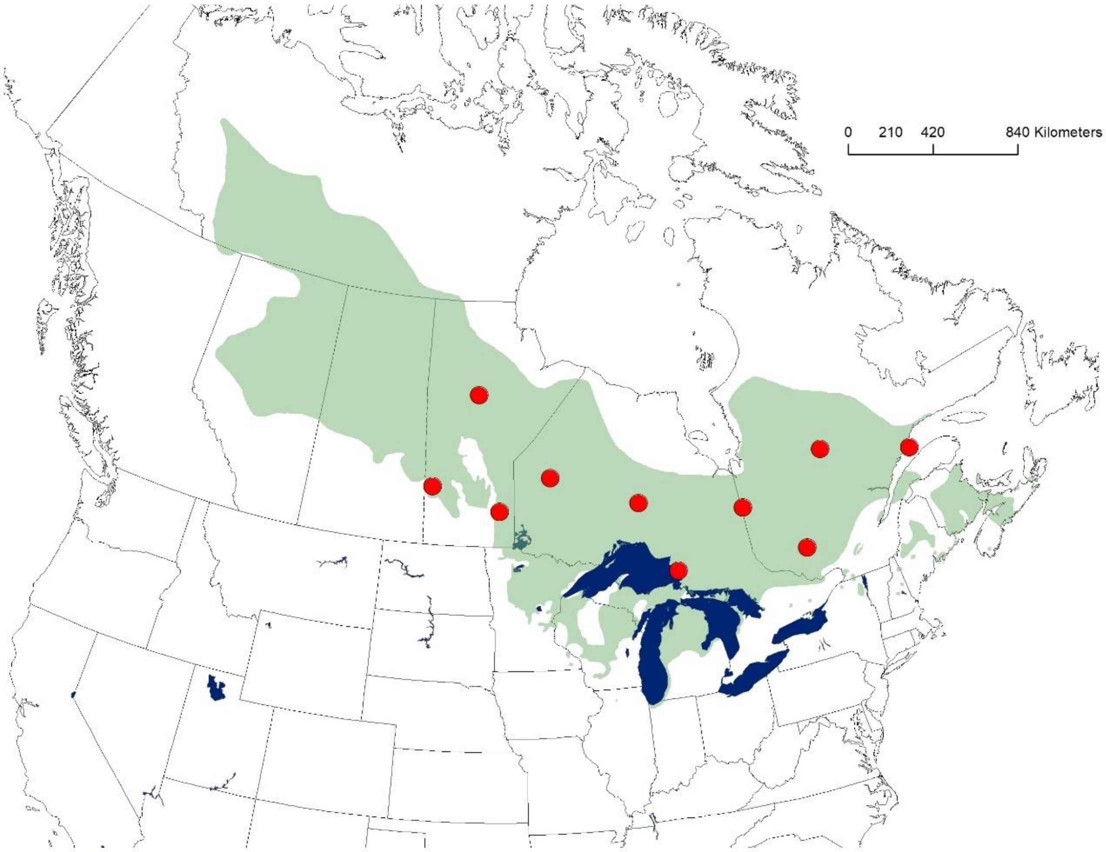

Fig S1.4 *Abies balsamea*

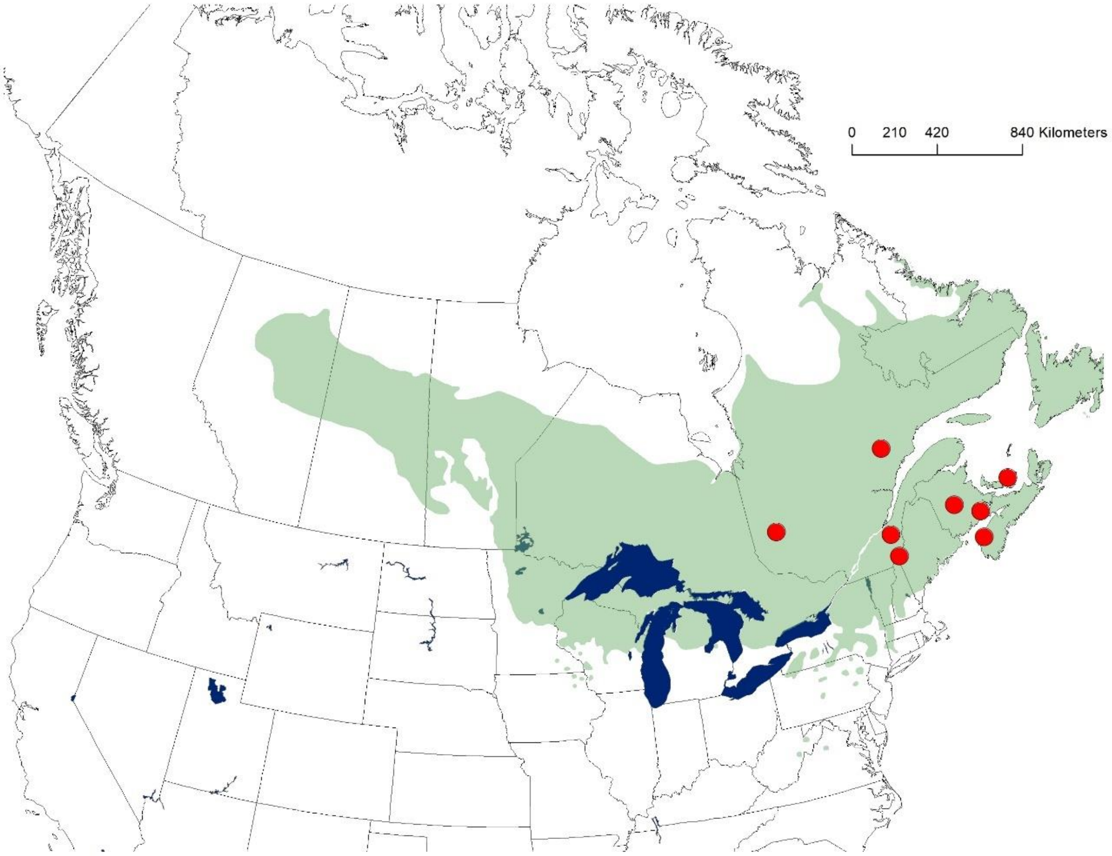

Fig S1.5 *Larix laricina*

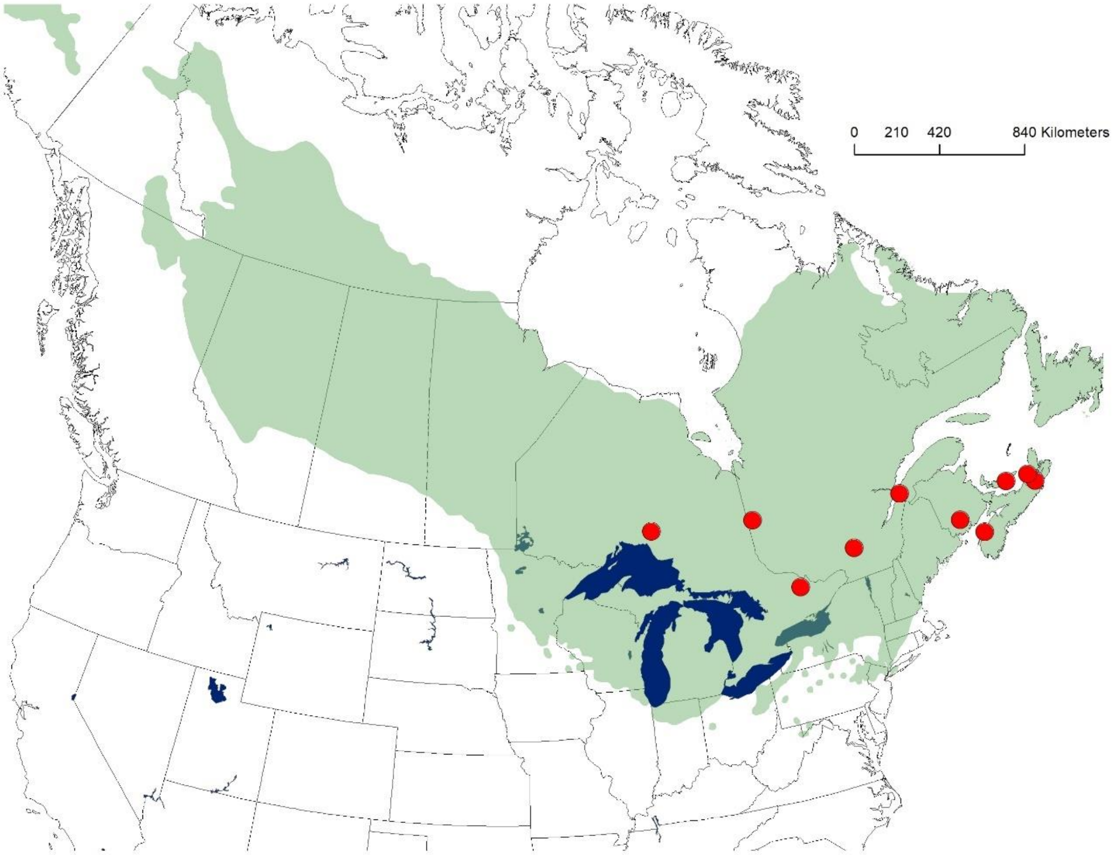

Fig S1.6 *Pinus strobus*

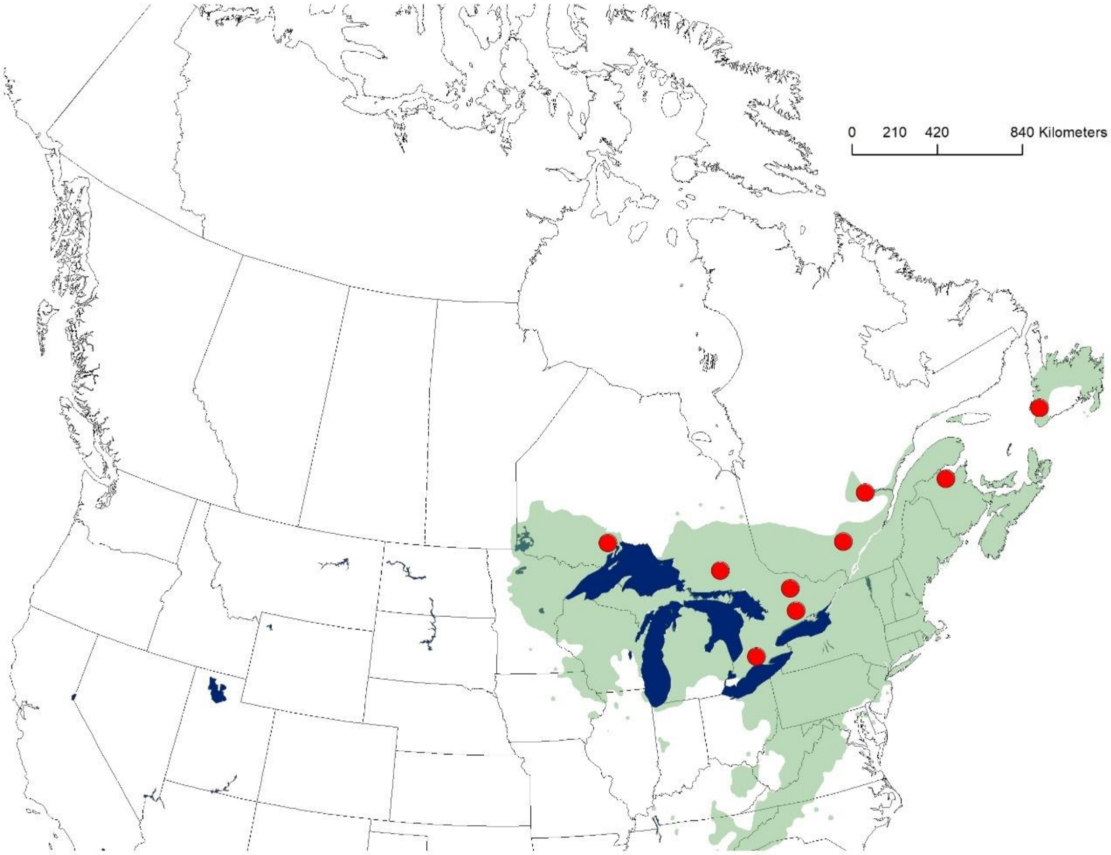

Fig S1.7 *Thuja occidentalis*

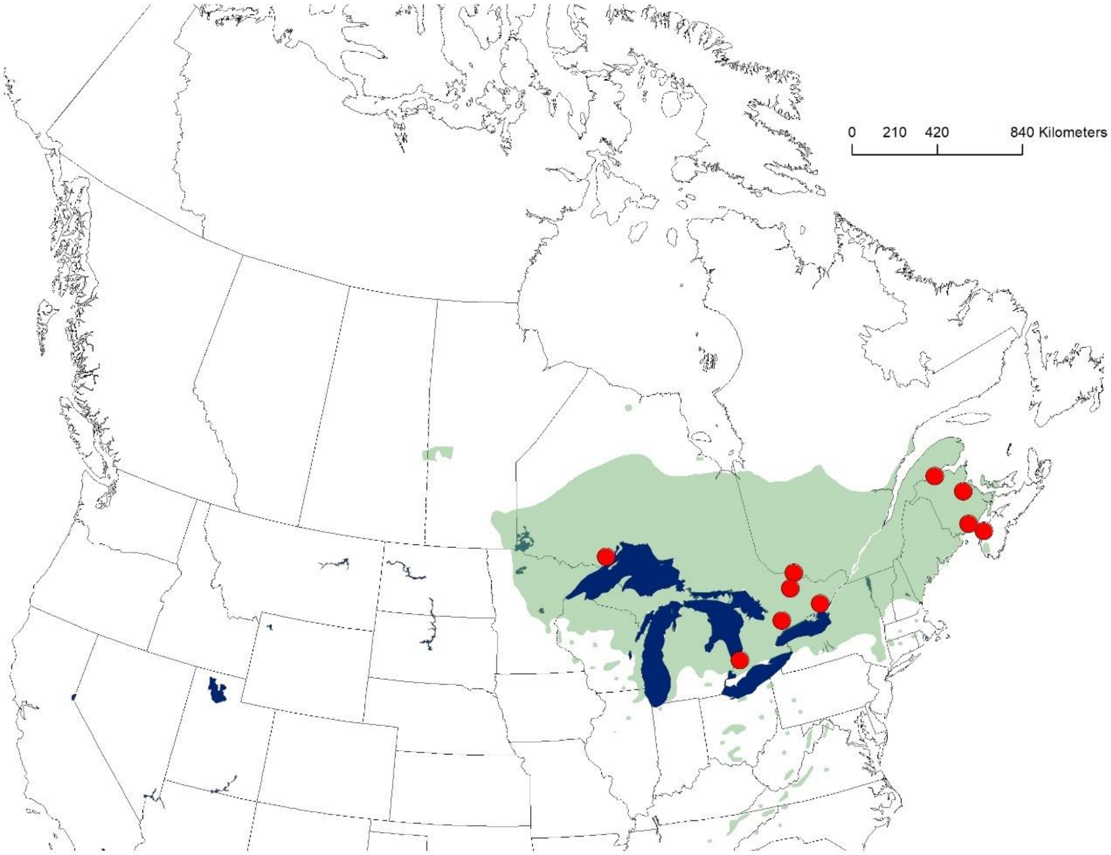

Methods S2. Production of sequence data

Seeds were first stratified during two months at 4°C, and then transferred to a growth chamber for germination during nine days, under the following conditions (24 hours cycle): 16 hours at 30°C air temperature and 750 lux luminosity, followed by 8 hours at 20°C air temperature in total darkness. Air humidity was kept at its maximum throughout the procedure.

Embryos and megagametophytes were ground using a mortar and pestle, and total RNA was extracted using the MasterPure™ Plant RNA Purification kit (Epicenter, Madison, WI, USA). RNA concentrations were assessed using a Nanodrop ND-1000 (Thermo Scientific, Wilmington, DE, USA) and RNA integrity was verified with an Agilent Bioanalyzer 2100, using RNA nano chips (Agilent Technologies Inc., Santa Clara, CA, USA). For each species, RNA extractions from 15 or 16 embryos that yielded sufficient quantity (27-33 ng) of high-quality RNA were pooled together at equimolar concentrations (total RNA mass of 500 ng) to synthetize a single library. Independent libraries were also synthetized for each of the four megagametophytes (500 ng of RNA for each megagametophyte) extracted for each species. Kapa Stranded mRNA-Seq kit version 3.15 (Kapa Biosystems, Wilmington, Massachusetts, USA) was used to synthetized mRNA libraries following the manufacturer's instructions with the following modifications: 1) fragmentation was performed at 94°C for 6 minutes in order to obtain insert size between 200-300 base pairs, and 2) concentrations of Illumina custom adapters (IDT, Coralville, Iowa, USA) was reduced by half at 25nM per reaction, in order to avoid adapter dimer molecules. The Axygen® AxyPrep™ Mag PCR Clean-Up Kit (Axygen Biosciences, Union City, CA, USA) was then used to purify DNA, and library quantification was performed using a Nanodrop ND-1000 (Thermo Scientific, Wilmington, DE, USA). The distribution of fragment size in libraries and the absence of adapter dimer molecules were assessed using an Agilent Bioanalyzer 2100 and High Sensitivity DNA chips (Agilent Technologies Inc., Santa Clara, CA, USA). At the sequencing step, one pool was prepared by merging libraries from single megagametophytes, and another one was prepared with the embryos. Each individual embryo from the pool and each single megagametophyte were at equimolar concentrations in the final mix. Both pools were purified a second time, and their concentration, as well as fragment size distribution, were evaluated with a Nanodrop ND-1000 and an Agilent Bioanalyzer 2100, respectively. Both pools were sequenced in paired-end mode (2×125 bp) with an Illumina HiSeq 2500 on one lane at the Genome Quebec Innovation Centre at McGill University (Montreal, Quebec, Canada).

Methods S3. Representation of the analyzed transcriptomes

Reference transcriptome sequences for the seven conifer species analyzed were obtained using a standardized RNA-seq protocol and released by Van Ghelder et al. (2019). In the sequence transcripts, open reading frames (ORF) were predicted with GeneMarkS-T version 5.1, using the default parameters (on the direct strand). This software was chosen for its proven accuracy in predicting genes predictions from transcriptome data, including in plants such as *Arabidopsis spp.* (Tang et al., 2015; http://exon.gatech.edu/GeneMark/).
In order to assess gene representation among species, transcript sequences overlapping an ORF and obtained for the six Pinaceae species were blasted against the most complete conifer transcriptome currently available for this family (Rigault et al., 2011). This reference transcriptome was developed in *Picea glauca* and contains 27,720 unique transcripts including above 23k full-length insert cDNAs (Rigault et al., 2011). Blastn version 2.13.0 program yielded above 60% of positive matches for both *Picea* species, and above 55% for the two *Pinus* species, as well as *Abies balsamea* and *Larix laricina* (sequence identify >70% at the nucleic level). Given that the Pinaceae and the Cupressaceae families diverged ~315 Mya (Leslie et al., 2018), *Thuja occidentalis* sequences were blasted against the red cedar (*Thuja plicata*) genome assembly recently published by Shalev et al. (2022). In total, 60.3% of the 39k *Thuja plicata* transcripts matched a *Thuja occidentalis* sequence analyzed in our study (sequence identify >70% at the nucleic level). Altogether, these results indicate that gene representation was homogeneous among the seven conifer species.

Methods S4. Preprocessing of raw RNAseq data

The quality of paired-end raw sequences was first assessed using FastQC version 0.11.2 (Andrews, 2010). The software SortMeRNA version 2.1 was then used for filtering rRNA fragments (Kopylova et al., 2012). Adapters, PCR primers and poor-quality sequences were removed using Trimmomatic version 0.36, using the settings recommended in Bolger et al. (2014). Finally, the quality of cleaned sequences was reassessed using FastQC to validate the quality control procedure. Filtered reads were aligned to a reference transcriptome using the bwa aln option implemented in the Burrows Wheeler Aligner (BWA) software version 0.7.13 (Li and Durbin, 2009) and the following command line: bwa aln –n 0.03 –t 2 your_reference_fasta_file.fa your_trimmomatic_output_file.fq > your_output_file.sai. Aligned bam files were sorted and indexed with SAMtools version 1.3 (Li et al., 2009), and duplicates were removed with PICARD version 2.0.1 (https://broadinstitute.github.io/picard/).

Methods S5. SNP calling

The Genome Analysis Toolkit HaplotypeCaller module was used for variants calling in each library (McKenna et al., 2010). Several filters were applied to retain only high-quality SNPs:

i- discard SNP clusters, that is when more than 3 SNPs were identify within a window of 35 bp

ii- filter out SNPs with QD<2.0 (variant confidence divided by the unfiltered depth <2), or with FS >60.0 (Phred-scaled p-value using Fisher's Exact Test to detect strand bias (the variation being seen on only the forward or only the reverse strand) in the reads), or with MQ <40.0 (Root Mean Square of the mapping quality of the reads), or with MQRankSum <-12.5 (This is the u-based z-approximation from the Mann-Whitney Rank Sum Test for mapping qualities (reads with ref bases vs. those with the alternate allele)), or with ReadPosRankSum < -8.0 (This is the u-based z-approximation from the Mann-Whitney Rank Sum Test for the distance from the end of the read for reads with the alternate allele.)

iii- retain only SNPs at positions with an alignment depth ≥10;

iv- retain only biallelic and non-singleton SNPs (i.e. reference and alternative alleles each supported by at least two reads);

v- retain only SNPs if both the reference allele and the alternative allele were observed in at least one diploid heterozygous sample.

These filtering steps were applied for SNPs called in pools of embryos, as well as for single megagametophytes.

Table S5.1 Metrics for the number of detected SNPs in the seven conifer species studied.

Species	Raw SNPs identified in pools of diploid embryos	High-quality SNPs identified in pools of diploid embryos (% of the number of raw SNPs)	High-quality SNPs identified in pools of diploid embryos, after removal of SNPs identified in haploid megagametophytes (% of the number of raw SNPs)
Picea glauca	243,309	151,376 (62.2%)	141,828 (58.3%)
Picea mariana	297,319	173,972 (58.5%)	160,129 (53.9%)
Pinus banksiana	203,541	129,286 (63.5%)	122,551 (60.2%)
Pinus strobus	168,250	103,502 (61.5%)	96,825 (57.5%)
Abies balsamea	187,400	130,897 (69.8%)	121,024 (64.6%)
Larix laricina	216,263	139,175 (64.4%)	135,103 (62.5%)
Thuja occidentalis	141,259	94,942 (67.2%)	89,102 (63.1%)
Total	1,457,341	923,150 (63.3%)	866,562 (59.5%)

Methods S6. Distributions of the lengths and depths of transcripts

The length and coverage (i.e. read depth) of transcripts were heterogenous across the seven conifer species investigated. Table S6.1 provides general statistics about the transcripts carrying SNP(s). The distributions of transcript length and depth showed differences across the seven species. For instance, *Pinus banksiana* and *Picea glauca* had the lowest and highest median transcript length, respectively (Table S6.1), while *Pinus banksiana* and *Abies balsamea* had the lowest and highest median transcript coverage, respectively. Transcript depth also differed across species, with *Pinus banksiana* showing the lowest median depth, while *Abies balsamea* had the highest value (Table S6.1). The Kolmogorov Smirnov test and the Cramer von Mises test confirmed that these distributions were heterogenous among some species for length and depth.

Table S6.1 General statistics about the analyzed transcripts carrying SNPs (length ≥ 300nt; mean depth ≥ 10; with an ORF predicted by GeneMark ST; with one SNP or more) for the seven conifer species studied.

Species	Number of transcripts carrying SNPs	Mean depth	Median depth	Cumulated length of transcripts (nt)	Mean length (nt)	Median length (nt)
Picea glauca	16,602	147	69	27 108 097	1633	1671
Picea mariana	18,601	134	62	29 299 382	1575	1296
Pinus banksiana	16,504	112	53	24 719 933	1498	1227
Pinus strobus	15,472	147	64	24 490 042	1583	1322
Abies balsamea	16,555	157	76	26 817 451	1620	1345
Larix laricina	17,014	151	66	28 496 392	1675	1391
Thuja occidentalis	13,892	133	66	23 973 403	1726	1468
Total/Average	114,640	140	65	184 904 700	1616	1389

Methods S7. Estimation of SNP abundance in transcripts

The raw number of SNPs appeared significantly correlated with transcripts length and depth in all seven conifer species, given that positive and negative correlations were observed with transcripts length and depth, respectively (Table S7.1). Correlations were rather weak according to Cohen's guidelines (Cohen, 1988). However, this artifact needed to be corrected before any statistical comparison between the datasets (Eo and DeWoody, 2012). Therefore, for each transcript, the number of SNPs was related to the transcript length and transcript depth though a negative binomial model (Eo and DeWoody, 2012). The model considered two variables (transcript length (L) and transcript depth (D)), and was applied sequentially to the seven conifer species. Only data derived from transcripts carrying at least one SNP were included for the modelling step, which considered a total 114,640 transcripts, such as:

$$\log(SNPs + 0.5) = \alpha_0 + \alpha_1 L + \alpha_2 D \quad (1)$$

where α_0, α_1, α_2 are the model's parameters for each transcript. This model was adjusted to the data using the GENMOD procedure of SAS (SAS Institute inc., release 9.4, NC).

We then corrected the observed values of log(*SNPs+0.5)* by translating their values parallel to the regression line, using formula (1), until the mean length $\bar{L}$ and the mean depth $\bar{D}$ computed over all the transcripts. This translation corresponds to the following formula defining the corrected number of SNPs (noted SNPs corr), such as:

$$\log(SNPs\ corr + 0.5) = \log(SNPs + 0.5) - \widehat{\alpha_1}(L - \bar{L}) - \widehat{\alpha_2}(D - \bar{D}) \quad (2)$$

where $\hat{\alpha}_1$and $\hat{\alpha}_2$ are the estimates of the parameters α_1 and α_2.

The modeling step enabled a decrease of the correlation values between the number of SNPs and the length and depth of the transcripts (Table S7.1). Even if four correlations remained significant between the number of SNPs and transcript length (Table S7.1), all of them were weak (Table S7.1). Therefore, it was possible to compare the distributions of the numbers of SNPs across species. This comparison showed a significant heterogeneity in the number of SNPs across species based on the deviation from mean for both Kolmogorov Smirnov and Cramer-von Mises tests (Table S7.2).

Table S7.1 Pearson correlation coefficients between the number of SNPs (raw number or log of this number estimated by the adjustment model after standardization) and transcript length and depth in the seven conifer species studied.

Species	Correlation between the raw number of SNPs and transcript length	Correlation between the log of the standardized number of SNPs estimated by the model and transcript length	Correlation between the raw number of SNPs and transcript depth	Correlation between the log of the standardized number of SNPs estimated by the model and transcript depth
Abies balsamea	0.484**	-0.007	-0.079**	0.004
Pinus banksiana	0.417**	-0.044**	-0.050**	0.006
Picea glauca	0.501**	-0.014	-0.034**	0.003
Larix laricina	0.425**	-0.054**	-0.041**	0.011
Picea mariana	0.534**	-0.009	-0.044**	0.005
Pinus strobus	0.272**	-0.008**	-0.058**	0.016
Thuja occidentalis	0.347**	-0.059**	-0.050**	0.014

Correlation test p-value <0.05(*), <0.01 (**)

Table S7.2 Statistical tests comparing the distributions of the number of SNPs across the seven conifer species studied after correction for the sequence length and sequencing depth effects

			Kolmogorov-Smirnov test		Cramer-von Mises test
Overall SNP diversity	Species	Number of transcripts	Empirical distribution function at maximum	Deviation from mean at maximum	Summed deviation from mean
Highest	*Picea glauca*	16,602	0.217	-12.27	84.67
Highest	*Picea mariana*	18,601	0.215	-13.37	75.05
Intermediate	*Pinus banksiana*	16,504	0.300	-1.68	1.32
Intermediate	*Abies balsamea*	16,555	0.291	-2.77	3.19
Intermediate	*Larix laricina*	17,014	0.318	0.72	0.38
Lowest	*Pinus strobus*	15,472	0.455	17.71	124.59
Lowest	*Thuja occidentalis*	13,892	0.434	14.24	98.75

Methods S8. Estimation of gene SNP A/S values and significance of the excess of nonsynonymous SNPs

Open reading frames (ORF) were first predicted with GeneMarkS-T, using the default parameters (on the direct strand). This software was shown to provide accurate gene predictions from transcriptome data, including in plants such as *Arabidopsis spp.* (Tang et al., 2015; http://exon.gatech.edu/GeneMark/). Synonymous and nonsynonymous SNPs along the longest ORF for each trascript were annotated using an in-house script. Next, transcripts carrying exclusively non-coding sites (i.e 3' UTR and 5' UTR regions) were discarded, and the number of nonsynonymous and synonymous sites in each coding sequence was then calculated. The number of synonymous sites (Ls) was defined as the number of 4-fold degenerate sites plus one-third of the number of 2-fold degenerate sites. Similarly, the number of nonsynonymous sites (La) was defined as the number of nondegenerate sites plus two-thirds of the number of 2-fold degenerate sites.

The SNP A/S ratio is a ratio of two rates, A and S, and it was calculated for each ORF as the number of SNPs per nonsynonymous site (A) divided by the number of SNPs per synonymous site (S). A and S were estimated for each ORF after identifying nonsynonymous and synonymous sites and classifying observed SNPs in nonsynonymous and synonymous ones. An adjusted SNP A/S ratio was used to include ORFs with no synonymous SNPs following the empirical logit principle (Agresti, 2013):

$$Adj.SNP\ A/S\ ratio\ = \frac{NS'/\,(La+1)}{S'/\,(Ls+1)} \qquad (1)$$

with NS' = number of nonsynonymous SNPs+0.5 and S' = number of synonymous SNPs+0.5.

We then assessed the significance of the excess of nonsynonymous SNPs (i.e. high A/S ratios) in each transcript, as such excess can potentially arise from chance alone, especially in transcripts with low number of SNPs. To do so, we first estimated the probability that a random mutation occurring in an ORF results in a nonsynonymous or a synonymous SNP (nsSNP or sSNP). The probability of occurrence of A and S mutations was determined empirically based on our whole dataset (all SNPed transcripts with ORFs, for all seven species) using the following formulas:

P(A) = (total number of nsSNPs) / (total number of SNPs)
P(S) = (total number of sSNPs mutations) / (total number of SNPs)

P(A) and P(S) were almost equal, reaching 0.501 and 0.499 (Table S8.1; see supporting excel file), respectively, which translated into a global observed A/S ratio of 0.35. Next, we used a binomial law to derive the associated probability for each possible combination of nonsynonymous and synonymous mutations in transcripts carrying 1 (lowest number of SNPs per transcript found in our dataset) to 49 (highest number of SNPs per transcript found in our dataset) SNPs. The probabilities associated with each A/S combination are provided in Table S8.1. Transcript which had both an A/S ratios exceeding 1 and a probability of occurrence of its combination of nonsynonymous (A) and synonymous (S) mutations lower than 5% were considered under positive selection (Table S8.2).

Table S8.2 SNP A/S ratio metrics for seven conifer species

Species	SNPed transcripts	Transcripts with A/S > 1	Transcripts with A/S > 1 and $P < 0.05$ (%)			
			Number of transcripts	% of SNPed transcripts	Mean A/S	Median A/S
Abies balsamea	14,252	2,987	314	2.2	2.89	2.20
Pinus banksiana	14,242	2,914	300	2.1	2.86	2.21
Picea glauca	14,742	2,737	321	2.2	2.70	1.99
Larix laricina	14,160	3,094	351	2.2	2.89	2.15
Picea mariana	16,232	3,144	301	2.1	2.85	2.03
Pinus strobus	12,362	3,147	233	1.9	2.84	1.95
Thuja occidentalis	10,823	2,640	227	2.1	2.82	1.96

Methods S9. GO enrichment tests

Enrichment tests were conducted with the R package topGO (Alexa et al., 2006; https://bioconductor.org/packages/release/bioc/html/topGO.html), in order to identify GO terms enriched among annotations of the 2,047 positively selected genes. Tests were conducted independently for each conifer species. The background set consisted of all the genes for which a SNP A/S value could be calculated, and the test set consisted of selected genes. Methods implemented in topGO were used to assess the significance of a GO term enrichment based on its neighborhood (weight01 method, nodesize=5, Fisher's test). To summarize the Gene Ontology terms describing the sequences, semantically similar terms were clustered by using the software REduceVIsualizeGO with a medium allowed similarity parameter of 0.7 (Supek et al., 2011). Venn diagrams were generated using the R package Venn (Dusa, 2021).

Supplementary figures

Figure S1. PFAM families and Gene Ontology terms most represented in the 2,047 positively selected genes in the seven conifer species studied. (A) PFAM families represented by 5 genes or more. Only GO terms associated with 20 gene sequences or more are shown in the following categories: biological processes (B), molecular functions (C) or cellular components (D), whatever the species. X-axis represents the number of genes with the annotation presented on Y-axis.

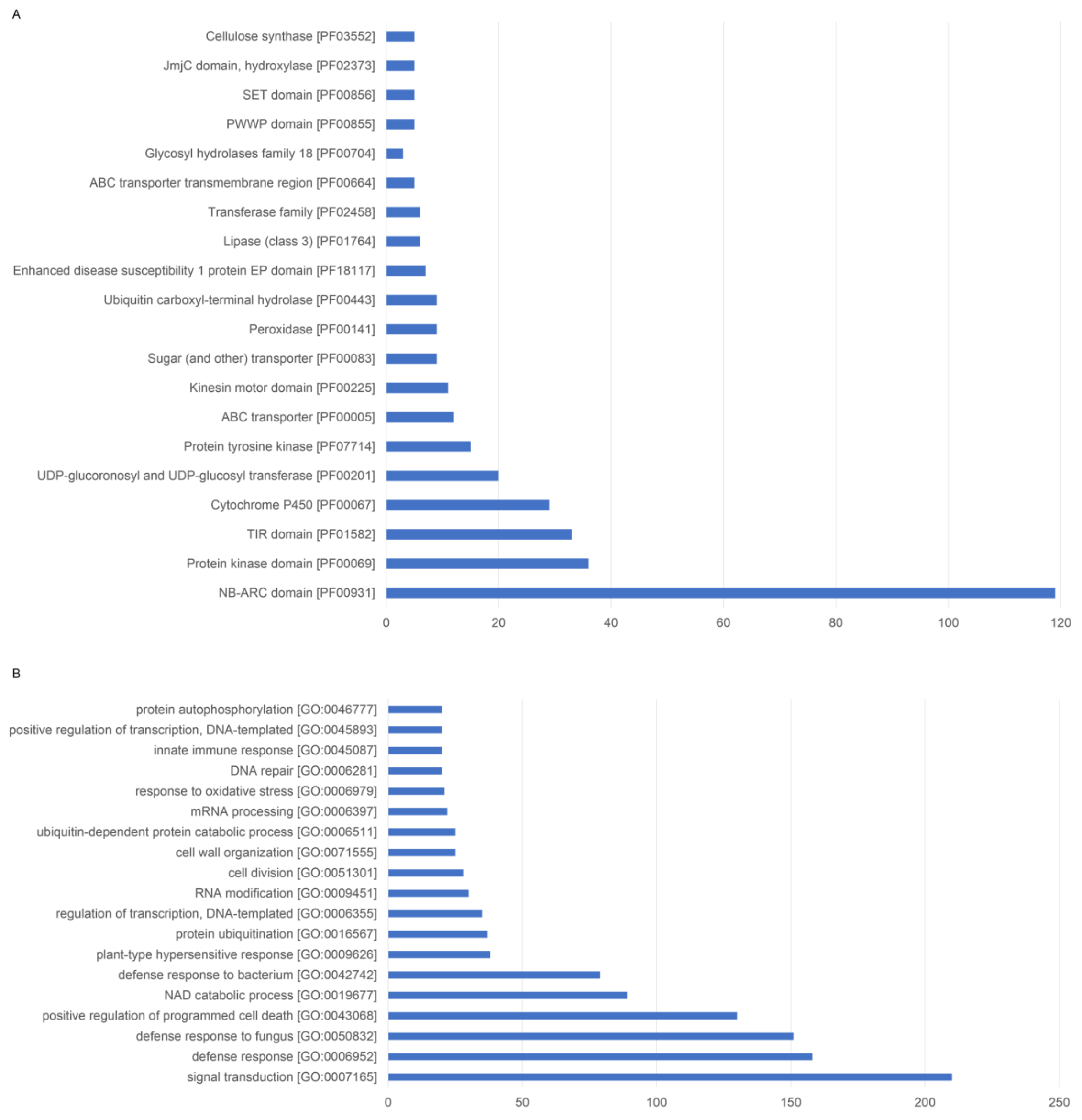

C

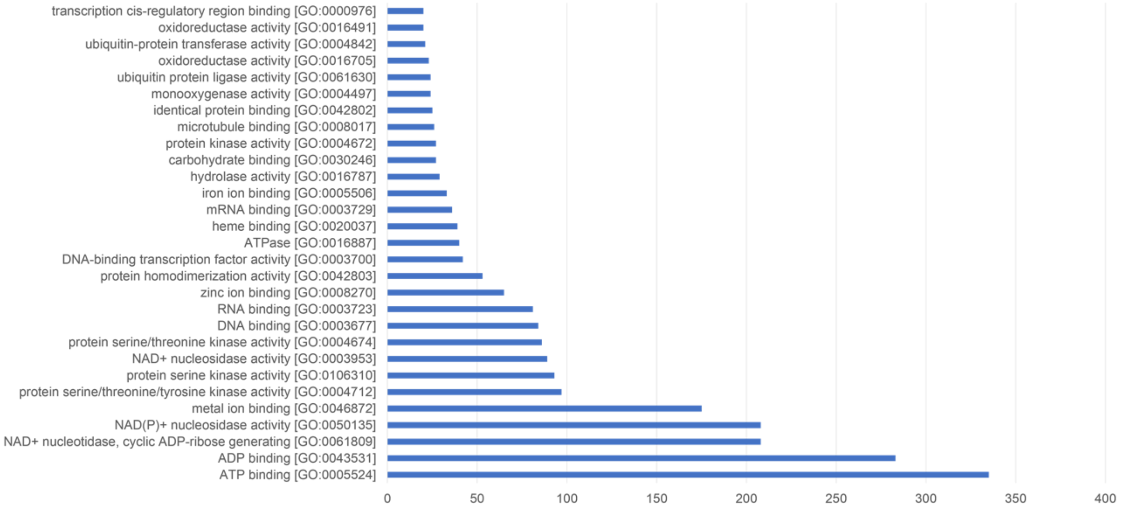
transcription cis-regulatory region binding [GO:0000976]
oxidoreductase activity [GO:0016491]
ubiquitin-protein transferase activity [GO:0004842]
oxidoreductase activity [GO:0016705]
ubiquitin protein ligase activity [GO:0061630]
monooxygenase activity [GO:0004497]
identical protein binding [GO:0042802]
microtubule binding [GO:0008017]
protein kinase activity [GO:0004672]
carbohydrate binding [GO:0030246]
hydrolase activity [GO:0016787]
iron ion binding [GO:0005506]
mRNA binding [GO:0003729]
heme binding [GO:0020037]
ATPase [GO:0016887]
DNA-binding transcription factor activity [GO:0003700]
protein homodimerization activity [GO:0042803]
zinc ion binding [GO:0008270]
RNA binding [GO:0003723]
DNA binding [GO:0003677]
protein serine/threonine kinase activity [GO:0004674]
NAD+ nucleosidase activity [GO:0003953]
protein serine kinase activity [GO:0106310]
protein serine/threonine/tyrosine kinase activity [GO:0004712]
metal ion binding [GO:0046872]
NAD(P)+ nucleosidase activity [GO:0050135]
NAD+ nucleotidase, cyclic ADP-ribose generating [GO:0061809]
ADP binding [GO:0043531]
ATP binding [GO:0005524]
0
50
100
150
200
250
300
350
400

D

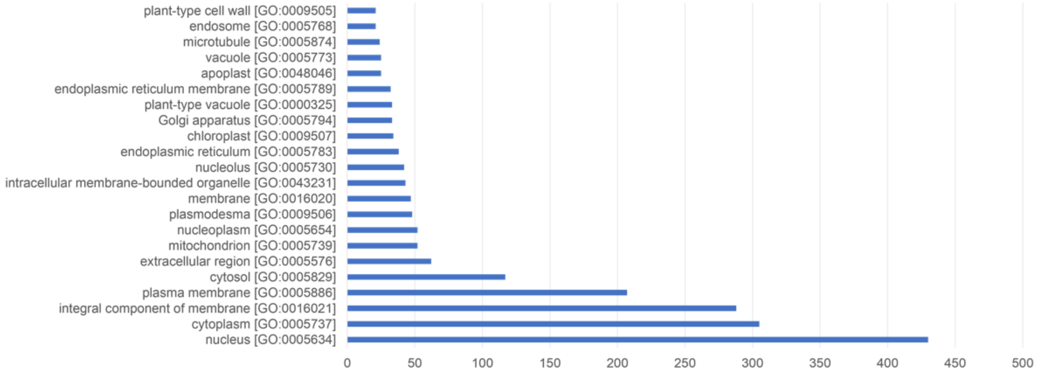
plant-type cell wall [GO:0009505]
endosome [GO:0005768]
microtubule [GO:0005874]
vacuole [GO:0005773]
apoplast [GO:0048046]
endoplasmic reticulum membrane [GO:0005789]
plant-type vacuole [GO:0000325]
Golgi apparatus [GO:0005794]
chloroplast [GO:0009507]
endoplasmic reticulum [GO:0005783]
nucleolus [GO:0005730]
intracellular membrane-bounded organelle [GO:0043231]
membrane [GO:0016020]
plasmodesma [GO:0009506]
nucleoplasm [GO:0005654]
mitochondrion [GO:0005739]
extracellular region [GO:0005576]
cytosol [GO:0005829]
plasma membrane [GO:0005886]
integral component of membrane [GO:0016021]
cytoplasm [GO:0005737]
nucleus [GO:0005634]
0
50
100
150
200
250
300
350
400
450
500

Figure S2. Heatmap showing the abundance of GO SLIM terms for biological processes across the seven conifer species studied in the overall sequence dataset and among positively selected genes in the species studied. (PG: *Picea glauca*, PM: *Picea mariana*, PB: *Pinus banksiana*, AB: *Abies balsamea*, LL: *Larix laricina*, TO: *Thuja occidentalis*, PS: *Pinus strobus*).

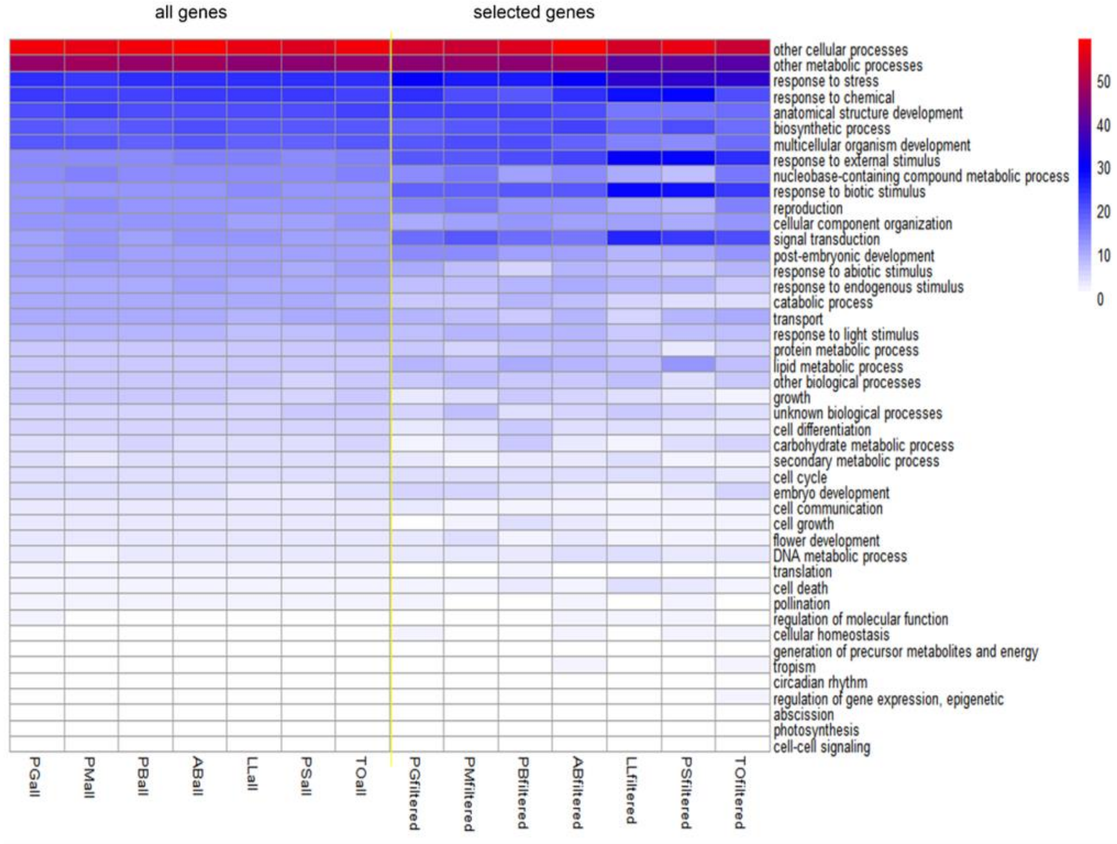

Figure S3. Heatmap showing the abundance of the 18 PFAM families shared at least by two conifer species, and represented five times or more among the annotations of the 2,047 positively selected genes. Each cell represents the number of genes found in one species (x-axis) and similar to a PFAM accession (y-axis) with an e-value < E-15. The data shown in each column are from *Picea glauca* (PG), *Picea mariana* (PM), *Pinus banksiana* (PB), *Abies balsamea* (AB), *Larix laricina* (LL), *Pinus strobus* (PS) and *Thuja occidentalis* (TO). The figure was drawn with the pheatmap package in R. The color scale on the right indicates the number of occurrences of each PFAM family.

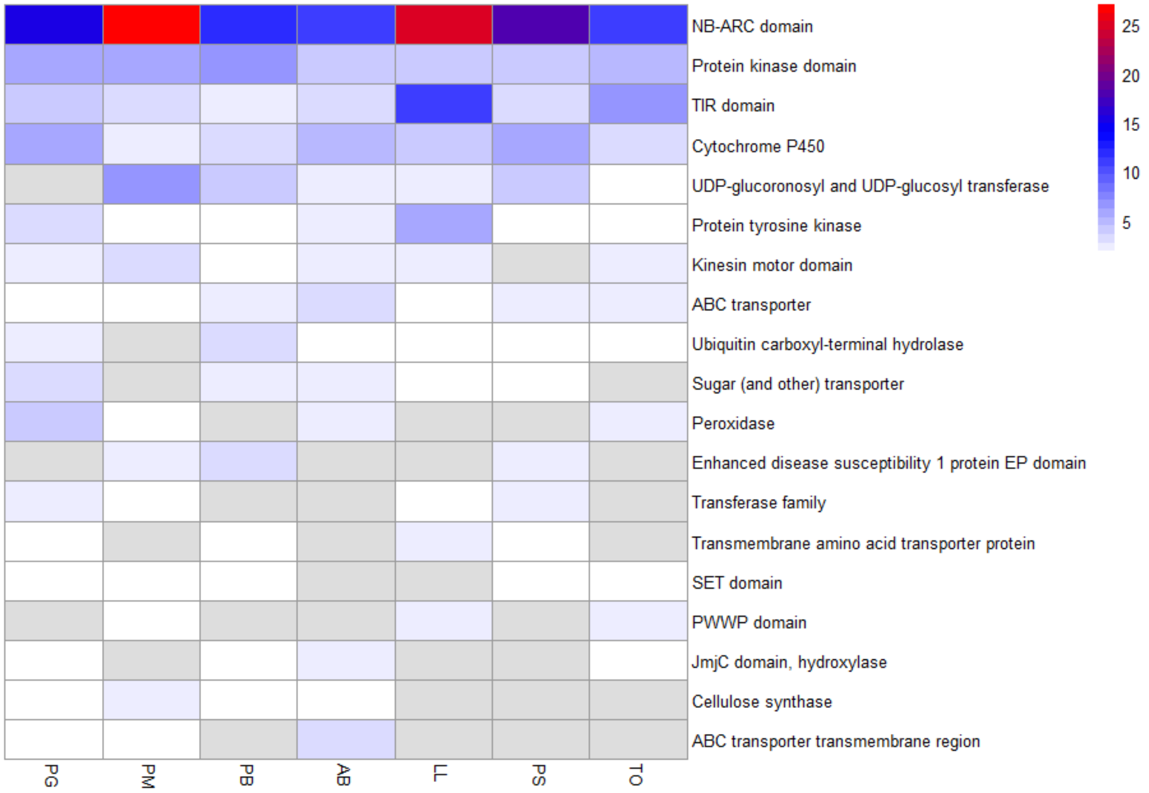

Figure S4. Categorization of the 29 biological processes associated with positively selected genes shared by all seven conifer species studied. Figure adapted from the REVIGO software for summarizing and visualizing lists of GO terms. Each rectangle represents a cluster of terms labeled with a representative term. Each color represents a supercluster identified based on a semantic similarity measure (Supek et al., 2011).

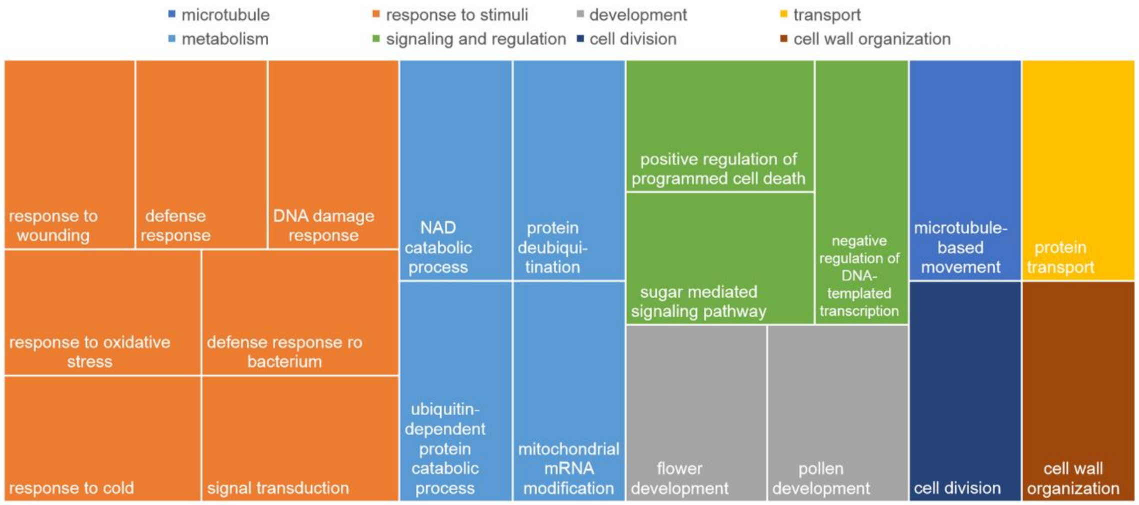

Figure S5. Abundance of the 64 biological processes represented 10 times or more among the annotations of the 2,047 positively selected genes. Each cell represents the number of genes found in one given species (x-axis) and annotated in a GO term (y-axis). The figure was drawn with the pheatmap package in R. The color scale on the right indicates the number of occurrences of each GO term.

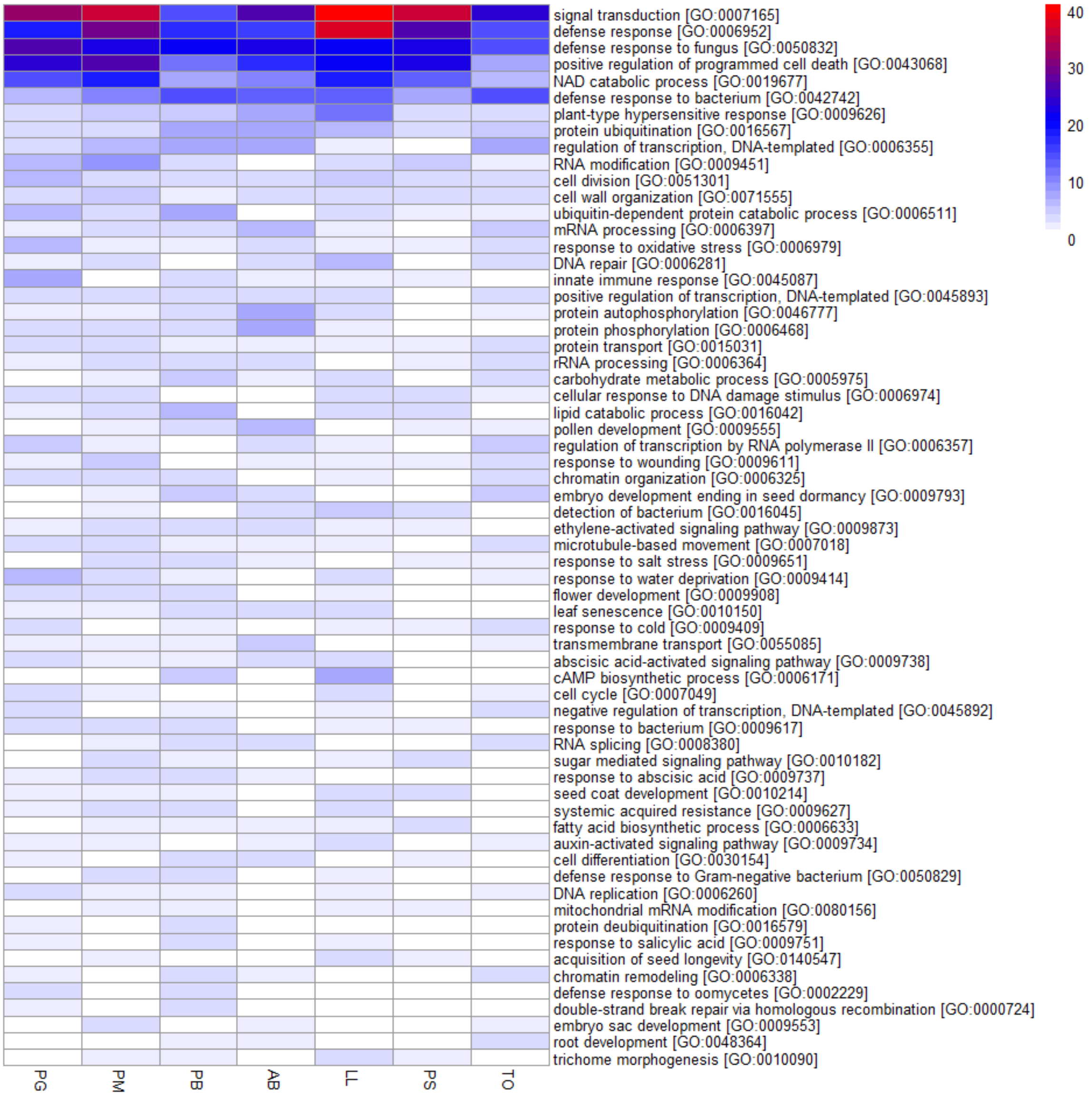

Supplementary Tables

Table S1. Metrics for high-quality SNPs detected in transcripts with open reading frames (ORFs) (transcript length ≥ 300nt; mean depth of read alignment ≥ 10; ORF predicted by GeneMark ST) for the seven conifer species studied. Numbers are based on raw SNPs data (without any adjustments for sequence length and read alignment depth).

Species	Transcripts with ORFs	Transcripts with SNP(s) (%)	ORFs with SNP(s) (%)	SNPs	Noncoding SNPs (% of SNPs)	Coding SNPs (% of SNPs)	Nonsynonymous SNPs (% of coding SNPs)	Synonymous SNPs (% of coding SNPs)
Picea glauca	18,060	16,602 (91.9)	14,742 (81.6)	105,778	37,590 (35.5)	68,188 (64.5)	34,001 (49.9)	34,187 (50.1)
Picea mariana	20,534	18,601 (90.6)	16,232 (79.0)	114,771	42,502 (37.0)	72,269 (63.0)	36,251 (50.2)	36,018 (49.8)
Pinus banksiana	19,510	16,504 (84.6)	14,242 (73.0)	90,985	32,447 (35.7)	58,538 (64.3)	29,361 (50.2)	29,177 (49.8)
Abies balsamea	19,487	16,555 (85.0)	14,252 (73.1)	91,311	33,189 (36.3)	58,122 (63.7)	29,669 (51.0)	28,453 (49.0)
Larix laricina	20,950	17,014 (81.2)	14,160 (67.6)	94,359	36,757 (39.0)	57,602 (61.0)	29,436 (51.1)	28,166 (48.9)
Pinus strobus	21,795	15,472 (71.0)	12,362 (56.7)	71,576	26,663 (37.3)	44,913 (62.7)	23,207 (51.7)	21,706 (48.3)
Thuja occidentalis	19,543	13,892 (71.1)	10,823 (55.4)	64,872	25,913 (39.9)	38,959 (60.1)	20,153 (51.7)	18,806 (48.3)
Total	139,879	114,640 (82.0)	96,813 (69.2)	633,652	235,061 (37.1)	398,591 (62.9)	202,078 (50.7)	196,513 (49.3)

Table S2. Kruskal-Wallis test showing significant differences in rates of total, synonymous and nonsynonymous SNPs across the seven conifer species studied.

Species	Average number of SNPs per 100 bp	Average number of synonymous SNPs per 100 bp	Average number of nonsynonymous SNPs per 100 bp
Picea glauca	0.00511	0.01007	0.00326
Picea mariana	0.00518	0.00989	0.00321
Pinus banksiana	0.00479	0.00961	0.00311
Abies balsamea	0.00439	0.00899	0.00300
Larix laricina	0.00442	0.00904	0.00295
Pinus strobus	0.00391	0.00852	0.00287
Thuja occidentalis	0.00358	0.00777	0.00254
Kruskal-Wallis test p-value	< 2.2e-16	< 2.2e-16	< 2.2e-16

Table S3. Metrics summarizing the content of the 16,982 orthogroups delineated with Orthofinder (Emms and Kelly, 2019) from a set of 139,889 genes from the seven conifer species studied.

Species	*Picea glauca*	*Picea mariana*	*Pinus banksiana*	*Abies balsamea*	*Larix laricina*	*Pinus strobus*	*Thuja occidentalis*	Total
Number of genes	18,060	20,534	19,510	19,487	20,950	21,795	19,553	139,889
Number of genes in orthogroups	17,514	19,631	18,536	18,609	19,742	20,382	18,004	132,418
Proportion of genes in orthogroups	97.0%	95.6%	95.0%	95.5%	94.2%	93.5%	92.1%	94.7%
Number of unassigned genes	546	903	974	878	1,208	1,413	1,549	7471
Proportion of unassigned genes	3.0%	4.4%	5.0%	4.5%	5.8%	6.5%	7.9%	5.3%
Number of orthogroups containing species	12,619	13,275	12,764	12,738	13,060	13,259	11,442	-
Proportion of orthogroups containing species	74.3%	78.2%	75.2%	75.0%	76.9%	78.1%	67.4%	-
Number of species-specific orthogroups	32	51	112	107	148	161	502	1,113
Number of genes in species-specific orthogroups	70	128	261	272	410	407	2,170	3,718
Proportion of genes in species-specific orthogroups	0.4%	0.6%	1.3%	1.4%	2.0%	1.9%	11.1%	2.7%
Number of species-specific orthogroups + unassigned genes	578	954	1,086	985	1,356	1,574	2,051	-
Number of genes in species-specific orthogroups + unassigned genes	616	1,031	1,235	1,150	1,618	1,820	3,719	-
Proportion of genes in species-specific orthogroups + unassigned genes	3.4%	5.0%	6.3%	5.9%	7.7%	8.4%	19.0%	-

Table S4. Pairwise proportions of shared orthogroups corresponding to positively selected genes between each pair of species. PG: white spruce (*Picea glauca*), PM: black spruce (*Picea mariana*), PB: jack pine (*Pinus banksiana*), AB: balsam fir (*Abies balsamea*), LL: tamarack (*Larix laricina*), PS: eastern white pine (*Pinus strobus*), TO: eastern white cedar (*Thuja occidentalis*).

Family	Taxa	PG	PM	PB	AB	LL	PS	TO	Average proportion of shared orthogroups
Pinaceae	PG								0.13
	PM	0.14							
	PB	0.15	0.14						
	AB	0.15	0.12	0.13					
	LL	0.15	0.12	0.14	0.14				
	PS	0.14	0.10	0.11	0.12	0.13			
Cupressaceae	TO	0.12	0.10	0.12	0.11	0.11	0.09		0.11

Table S5. Main families of disease resistance genes found in the 2,047 positively selected conifer genes.

Gene family	Accession of homologous protein	Recognotion or resistance regulation against	Number of positively selected genes	Species[1]	Match a PSG[2] in *Brassica* spp. or poplar	Function
RUN1	RUN1_VITRO	Mildew	72	PG, PM, PB, LL, AB, PS, TO	No	Disease resistance (R) protein that confers resistance to multiple powdery and downy mildew by promoting cell death (PubMed:24033846, PubMed:31439792). Acts as a NAD^+ hydrolase (NADase): in response to activation, catalyzes cleavage of NAD^+ into ADP-D-ribose (ADPR) and nicotinamide; NAD^+ cleavage triggering a defense system that promotes cell death (PubMed:31439792). Also able to hydrolyze $NADP^+$, but not other NAD^+-related molecules (PubMed:31439792).
RPV1	RPV1_VITRO	Powdery and downy mildew	41	PG, PM, PB, AB, LL, PS, TO	No	Acts as a NAD^+ hydrolase (NADase): in response to activation, catalyzes cleavage of NAD^+ into ADP-D-ribose (ADPR) and nicotinamide; NAD^+ cleavage triggering a defense system that promotes cell death
DLO1	DLO1_ARATH	Negative regulator of defense against Hyaloperonospora arabidopsidis	3	PG, PB	Yes	Component of a negative feedback regulation system of SA levels during senescence. Regulates both onset and progression of leaf senescence (PubMed:23959884). Negative regulator of defense against Hyaloperonospora arabidopsidis (PubMed:25376907).Confers susceptibility to the downy mildew pathogen Hyaloperonospora arabidopsidis
ROQ1	ROQ1_NICBE	*Xanthomonas* and *Pseudomonas syringae*	41	PG, PM, PB, LL, AB, PS, TO	No	Acts as a NAD^+ hydrolase (NADase): in response to activation, catalyzes cleavage of NAD^+ into ADP-D-ribose (ADPR) and nicotinamide;

						NAD^+ cleavage triggering a defense system that promotes cell death
L6	L6_LINUS	Rust	15	PG, PM, LL,	No	Acts as a NAD^+ hydrolase (NADase): in response to activation, catalyzes cleavage of NAD^+ into ADP-D-ribose (ADPR) and nicotinamide; NAD^+ cleavage triggering a defense system that promotes cell death
RPS2	RPS2_ARATH	*Pseudomonas syringae*	10	PM, LL, AB, PS	Yes	Disease resistance (R) protein that specifically recognizes the AvrRpt2 type III effector avirulence protein from *Pseudomonas syringae.* Resistance proteins guard the plant against pathogens that contain an appropriate avirulence protein via an indirect interaction with this avirulence protein. That triggers a defense system including the hypersensitive response, which restricts the pathogen growth. Acts via its interaction with RIN4, and probably triggers the plant resistance when RIN4 is degraded by AvrRpt2
RPS5	RPS5_ARATH	*Pseudomonas syringae*	5	PB, AB, LL, TO	Yes	Disease resistance (R) protein that specifically recognizes the avrPphB type III effector avirulence protein from *Pseudomonas syringae*. Also confers resistance against *Hyaloperonospora parasitica* (downy mildew). Resistance proteins guard the plant against pathogens that contain an appropriate avirulence protein via an indirect interaction with this avirulence protein. That triggers a defense system including the hypersensitive response, which restricts the pathogen growth. Requires PBS1 to trigger the defense reaction against avrPphB. In case of infection by *Pseudomonas syringae,* AvrPphB triggers RPS5-mediated defense mechanism via the cleavage of PBS1, suggesting that the cleavage of PBS1 could trigger an exchange of

						ADP for ATP, thereby activating RPS5. May function as a fine-tuned sensor of alterations in the structure of the effector target PBS1
RFL1 (RPS5-like)	RFL1_ARATH	*Pseudomonas syringae*	2	PG, PM	Yes	RPS5-like
TAO1	TAO1_ARATH	*Pseudomonas syringae*	23	PG, PM, PB, AB, LL, PS, TO	No	TIR-NB-LRR receptor-like protein that contributes to disease resistance induced by the Pseudomonas syringae type III effector AvrB. Acts additively with RPM1 to generate a full disease resistance response to *Pseudomonas syringae* expressing this type III effector
RPP3	R13L4_ARATH	*Pseudomonas syringae / Xanthomonas*	2	PM, TO	No	CC-NB-LRR receptor-like protein required for recognition of pathogenic bacteria type III effectors (T3E) such as *Pseudomonas syringae* HopZ1a and HopF2a and *Xanthomonas campestris pv. campestris* (Xcc) XopAC/AvrAC; this recognition requires ZED1-related kinases (e.g. PBL2, ZRK3 and ZED1/ZRK5) (PubMed:20368970, PubMed:26355215, PubMed:28288096, PubMed:30948527, PubMed:30948526, PubMed:28652264). Confers allele-specific recognition and virulence attenuation of HopZ1a (PubMed:20368970). Immunity mediated by RPP13L4/ZAR1 is independent of several genes required by other resistance protein signaling pathways such as NDR1 and RAR1 (PubMed:20368970). Together with ZED1/ZRK5, involved in the regulation of the ambient temperature-sensitive intersection of growth and immune response in the absence of pathogens (PubMed:28499073)
RPP1	RPP1_ARATH	*Hyaloperonospora (mildew)*	1	PB	No	TIR-NB-LRR receptor-like protein that confers resistance to the pathogen *Hyaloperonospora arabidopsis* (by similarity).

						Probably acts as a NAD^+ hydrolase (NADase): in response to activation, catalyzes cleavage of NAD^+ into ADP-D-ribose (ADPR) and nicotinamide; NAD^+ cleavage triggering a defense system that promotes cell death (PubMed:31439792, PubMed:31439793)
GLR33	GLR33_ARATH	*Hyaloperonospora (mildew)*	4		No	Glutamate receptor 3.3, Contributes to pathogen-associated molecular patterns (PAMP) elicitor-mediated resistance (PubMed:23952652)., Inovlved in resistance against Hyaloperonospora arabidopsidis (PubMed:23952652). Required for glutathione-induced defense responses, and innate immunity responses against the bacterial pathogen Pseudomonas syringae pv tomato strain DC3000 (PubMed:23656893).
EML3	EML3_ARATH		1	AB	No	EMSY-like genes are required for full RPP7-mediated race-specific immunity and basal defense in Arabidopsis.(Pubmed: 21830950)
RLK7	RLK7_ARATH		5	PG, PB, AB, PS	Yes	Receptor-like protein kinase 7; Plays a role in pattern-triggered immunity (PTI) signaling induced by pathogen-associated molecular patterns (PAMPs). Acts as a receptor for PIP1 defense peptide. PIP1 is an endogenous secreted peptide that acts as elicitor of immune response and positive regulator of defense response (PubMed:25188390)
EFR	EFR_ARATH		4	AB, LL	Yes	Constitutes the pattern-recognition receptor (PPR) that determines the specific perception of elongation factor Tu (EF-Tu), a potent elicitor of the defense response to pathogen-associated molecular patterns (PAMPs); phosphorylates BIK1 upon elicitation to regulate immune responses such as defense hormone expression (e.g. jasmonic acid (JA) and salicylic acid (SA)) (PubMed:29649442).

FLS2	FLS2_ARATH	bacteria	2	AB, PS	Yes	Constitutes the pattern-recognition receptor (PPR) that determines the specific perception of flagellin (flg22), a potent elicitor of the defense response to pathogen-associated molecular patterns (PAMPs). Flagellin-binding to the receptor is the first step to initiate the innate immune MAP kinase signaling cascade (MEKK1, MKK4/MKK5 and MPK3/MPK6), resulting in enhanced resistance against pathogens. Binding to the effector AvrPto1 or to the phosphatase hopD2 from Pseudomonas syringae blocks the downstream plant immune response.
MLO6	MLO6_ARATH	powdery mildew fungus	1	PS	No	
LR10	LRL11_ARATH<br>LRL12_ARATH	Leaf rust	1	LL	Yes	
UNI	UNI_ARATH	Not applicable	2	PG, AB	Yes	Involved in disease resistance via the salicylic acid (SA) signaling pathway (PubMed:18315541, PubMed:27016096). Involved in shoot architecture development via the cytokinin signaling pathway (PubMed:18315541, PubMed:27016096)
SUMM2	SUMM2_ARATH	*Pseudomonas syringae*<br><br>*Hyaloperonospora arabidopsidis*	2	PG, PB, AB	Yes	Functions downstream of MEKK2/SUMM1 in immune responses, including cell death and defense responses<br>Negatively regulated by the MEKK1-MKK1-MKK2-MPK4 kinase cascade
RGA3	RGA3_SOLBU	Blight	3	AB, LL, TO	Yes	Belongs to a four-gene family located at the same locus. Although the four genes are expressed in the resistant haplotype, only RGA2 confers the resistance to *P.infestans*. In the susceptible haplotype, RGA1 and RGA3 are likely to be

						pseudogenes created by deletions and mutations, while RGA2 contains also several modifications
LRKS4	LRKS4_ARATH	*Phytophtora, Pseudomonas*	2	PB	Yes	L-type lectin-domain containing receptor kinase, Involved in resistance response to the pathogenic oomycetes Phytophthora infestans and Phytophthora capsici and to the pathogenic bacteria Pseudomonas syringae.
LRKS7	LRKS7_ARATH	*Phytophtora*	2	PM, TO	Yes	L-type lectin-domain containing receptor kinase, Involved in resistance response to the pathogenic oomycetes Phytophthora infestans and Phytophthora capsici
TMVRN	TMVRN_NICGU	TMV	11	PG, PM, AB, LL, TO	No	Resistance proteins guard the plant against pathogens that contain an appropriate avirulence protein via a direct or indirect interaction with this avirulence protein. That triggers a defense system including the hypersensitive response, which restricts the pathogen growth
RPP8-like protein 4	RP8L4_ARATH	turnip crinkle virus	1	PB	Yes	Disease resistance protein (CC-NBS-LRR class) family; The interaction with TIP (TCV-interacting protein) may be essential for the recognition of the avirulence proteins, and the triggering of the defense response. Triggers resistance to turnip crinkle virus (TCV) via a SAG101-dependent pathway
EDS1	EDS1C_ARATH	turnip crinkle virus	3	PG, PB, LL	No	Positive regulator of basal resistance and of effector-triggered immunity specifically mediated by TIR-NB-LRR (TNL) resistance proteins.
EDS1B	EDSBC_ARATH	turnip crinkle virus	3	PB, PS	No	Acts as a second functional copy of EDS1. Can mediate HRT-mediated resistance to turnip crinkle virus
EDS1L	EDS1L_ARATH		1	TO	No	Positive regulator of basal resistance and of effector-triggered immunity specifically mediated by TIR-NB-LRR resistance proteins. Disruption by bacterial effector of EDS1-TIR-NB-LRR resistance protein interactions constitutes the first step in resistance activation (PubMed:22158819).

TIR1	TIR1_ARATH		1	PM	No	Resistance proteins guard the plant against pathogens that contain an appropriate avirulence protein via a direct or indirect interaction with this avirulence protein. That triggers a defense system including the hypersensitive response, which restricts the pathogen growth (By similarity)
TBL44	TBL44_ARATH	powdery mildew	1	LL	No	Required for nonhost resistance (NHR) during plant-microbe interactions. Plants mutated in PMR5 are resistant to powdery mildew species (PubMed:15584961, PubMed:19810803).
EIX2	EIX2_SOLLC	*Trichoderma viride*	5	PB, TO	Yes	ethylene-inducing xylanase (EIX), involved in plant defense. Confers resistance to the fungal pathogen T.viride through recognition of the EIX elicitor protein

[1] *Abies balsamea* (AB), *Larix laricina* (LL), *Pinus banksiana* (PB), *Picea glauca* (PG), *Picea mariana* (PM), *Pinus strobus* (PS), *Thuja occidentalis* (TO)

[2] Positively selected genes from Guo et al. (2017) and Lin et al. (2018)

Table S6. Main families of genes in the chitin pathway with a role in defense against pathogens among the 2,047 positively selected conifer genes.

Gene family	Accession of homologous protein	Recognotion or resistance regulation against	Number of positively selected genes	Species[1]	Match a PSG in *Brassica* spp. or poplar	Function
CERK1	CERK1_ORYSA	pathogenic fungi, detection of microbial peptidoglycans	1	PG	Yes	Lysin motif (LysM) receptor kinase required as a cell surface receptor for chitin elicitor (chitooligosaccharides) signaling leading to innate immunity in response to biotic stresses. Involved in the resistance to pathogenic fungi, probably by sensing microbe-associated molecular patterns (MAMP) and pathogen-associated molecular patterns (PAMP) (PubMed:21070404, PubMed:22891159, PubMed:24964058).
CHIT2	CHIT2_TULSB	chitin containing fungal pathogens		PB, LL, TO	No	Defense against chitin containing fungal pathogens.
LYK5	LYK5_ARATH	Fungi		PG	Yes	The kinase LYK5 is a major chitin receptor in Arabidopsis and forms a chitin-induced complex with related kinase CERK1.(PMID: 25340959)

[1] *Abies balsamea* (AB), *Larix laricina* (LL), *Pinus banksiana* (PB), *Picea glauca* (PG), *Picea mariana* (PM), *Pinus strobus* (PS), *Thuja occidentalis* (TO)

Table S7. Main families of genes in the secondary metabolites pathway with a role in defense against pathogenes among the 2,047 positively selected conifer genes.

Gene family	Accession of homologous protein	Resistance against	Number of positively selected genes	Species[1]	Match a PSG in *Brassica* spp. or poplar	Function
Cytochrome P450 76T24	CYT24_CATRO	insects	4	PG, AB, LL, PS	No	Synthesis of monoterpenoid indole alkaloids
Abietadienol/abietadienal oxidase	C72B1_PINTA	herbivores	2	PG, PS	No	Formation of a diverse suite of diterpene resin acids defense metabolites
Delta-selinene synthase	TPSD4_ABIGR	insects	1	PS	No	Defensive oleoresin formation in conifers in response to insect attack or other injury

Table S8. Biological processes (associated to five genes or more) describing the 384 genes under positive selection in conifers and homologous to positively selected genes in poplar or *Arabidopsis thaliana* (Guo et al. 2017; Lin et al. 2018). The homologs were identified after a blastp search of the 2,047 positively selected conifer genes against the poplar genes and the *Arabidopsis* genes with a *Ka/Ks* >1.

Category	Identifier	Name	Number of genes
Stress reponses			
	GO:0006952	defense response	53
	GO:0042742	defense response to bacterium	20
	GO:0009626	plant-type hypersensitive response	16
	GO:0016045	detection of bacterium	15
	GO:0006979	response to oxidative stress	14
	GO:0050832	defense response to fungus	13
	GO:0045087	innate immune response	9
	GO:0002229	defense response to oomycetes	7
	GO:0009409	response to cold	6
	GO:0002237	response to molecule of bacterial origin	6
	GO:0009617	response to bacterium	5
Metabolism			
	GO:0013310	phosphorylation	63
	GO:0046777	protein autophosphorylation	12
	GO:0016567	protein ubiquitination	11
	GO:0006508	proteolysis	9
	GO:0042744	hydrogen peroxide catabolic process	9
	GO:0016042	lipid catabolic process	8
	GO:0006486	protein glycosylation	5
	GO:0009813	flavonoid biosynthetic process	5
	GO:0016131	brassinosteroid metabolic process	5
Developmental processes			
	GO:0010214	seed coat development	11
	GO:0140547	acquisition of seed longevity	8
	GO:0009845	seed germination	5
Response to stimulus			
	GO:0010182	sugar mediated signaling pathway	9
	GO:0009741	Response to brassinosteroid	6
	GO:0009873	Ethylene-activated signaling pathway	6
Regulatory processes			
	GO:0006355	regulation of DNA-templated transcription	6
	GO:0045893	positive regulation of DNA-templated transcription	5
	GO:0010359	regulation of anion channel activity	6
Other processes			
	GO:0080156	mitochondrial mRNA modification	11
	GO:0071555	cell wall organization	9
	GO:0009451	RNA modification	9
	GO:0007018	microtubule-based movement	10

[1] *Abies balsamea* (AB), *Larix laricina* (LL), *Pinus banksiana* (PB), *Picea glauca* (PG), *Picea mariana* (PM), *Pinus strobus* (PS), *Thuja occidentalis* (TO)

REFERENCES FOR THE SUPPLEMENTARY MATERIAL

Agresti, A. (2013). Categorical Data Analysis. 3rd Edition, John Wiley and Sons Inc., Hoboken.

Alexa, A., Rahnenführer, J., and Lengauer, T. (2006) Improved scoring of functional groups from gene expression data by decorrelating GO graph structure. *Bioinformatics* 22, 1600-1607. DOI: 10.1093/bioinformatics/btl140

Andrews, S. (2010). FastQC: A Quality Control Tool for High Throughput Sequence Data [Online]. Available from: http://www.bioinformatics.babraham.ac.uk/projects/fastqc/

Bolger, A.M., Lohse, M., and Usadel, B. (2014). Trimmomatic: a flexible trimmer for illumina sequence data. *Bioinformatics* 30, 2114-2120. DOI: 10.1093/bioinformatics/btu170

Cohen J. (1988). Statistical Power Analysis for the Behavioral Sciences (2nd ed.). Hillsdale, NJ: Lawrence Erlbaum Associates, Publishers.

Dusa, A. (2021). Draw Venn Diagrams [R package venn version 1.10]. R Foundation for Statistical Computing: Vienna, Austria.

Emms, D.M., and Kelly, S. (2019). Orthofinder: phylogenetic orthology inference for comparative genomics. *Genome Biol.* 20, 238. DOI: 10.1186/s13059-019-1832-y

Eo, S.H., and DeWoody, J.A. (2012). The effects of contig length and depth on the estimation of SNP frequencies, and the relative abundance of SNPs in protein-coding and non-coding transcripts of tiger salamanders (*Ambystoma tigrinum*). *BMC Genomics* 13,1. DOI: 10.1186/1471-2164-13-259

Guo, Y., Liu, J., Zhang, J., Liu, S., and Du, J. (2017). Selective modes determine evolutionary rates, gene compactness and expression patterns in Brassica. *Plant J.* 91,34-44. DOI: 10.1111/tpj.13541

Kopylova, E., Noé, L., and Touzet, H. (2012). SortMeRNA: fast and accurate filtering of ribosomal RNAs in metatranscriptomic data. *Bioinformatics* 28, 3211-3217. DOI: 10.1093/bioinformatics/bts611

Leslie, A.B., Beaulieu, J., Holman, G., Campbell, C.S., Mei, W., Raubeson, L.R., et al. (2018). An overview of extant conifer evolution from the perspective of the fossil record. *Am. J. Bot.* 105,1531-1544. DOI: 10.1002/ajb2.1143

Li, H., and Durbin, R. (2009). Fast and accurate short read alignment with Burrows–Wheeler transform. *Bioinformatics* 25, 1754-1760. DOI: 10.1093/bioinformatics/btp324

Li, H., Handsaker, B., Wysoker, A., Fennell, T., Ruan, J., Homer, N., et al. (2009). The Sequence Alignment/Map format and SAMtools. *Bioinformatics* 25, 2078-2079. DOI: 10.1093/bioinformatics/btp352

Lin, Y.C., Wang, J., Delhomme, N., Schiffthaler, B., Sundström, G., Zuccolo, A., et al. (2018). Functional and evolutionary genomic inferences in *Populus* through genome and population sequencing of American and European aspen. *Proc. Natl. Acad. Sci. U.S.A.* 115, 10970-10978. DOI: 10.1073/pnas.1801437115

McKenna, A., Hanna, M., Banks, E., Sivachenko, A., Cibulskis, K., Kernytsky, A., et al*.* (2010). The Genome Analysis Toolkit: a MapReduce framework for analyzing next-generation DNA sequencing data. *Genome Res.* 20, 1297-1303. DOI: 10.1101/gr.107524.110

Rigault, P., Boyle, B., Lepage, P., Cooke, J.E., Bousquet, J., and MacKay, J.J. (2011). A white spruce gene catalog for conifer genome analyses. *Plant Phys.* 157, 14-28. DOI: 10.1104/pp.111.179663

Shalev, T.J., Gamal El-Dien, O., Yuen, M.M.S., Shengqiang, S., Jackman, S.D., Warren, R.L., et al*.* (2022). The western red cedar genome reveals low genetic diversity in a self-compatible conifer. *Genome Res.* 32, 1952-1964. DOI: 10.1101/gr.276358.121

Supek, F., Bošnjak, M., Škunca, N., and Šmuc, T. (2011). REVIGO summarizes and visualizes long lists of gene ontology terms. PLoS one 6, e21800. DOI: 10.1371/journal.pone.0021800

Tang, S., Lomsadze, A., and Borodovsky, M. (2015). Identification of protein coding regions in RNA transcripts. *Nuc. Acids Res.* 43, e78. DOI: 10.1093/nar/gkv227

Van Ghelder, C., Parent, G.J., Rigault, P., Prunier, J., Giguère, I., Caron, S., et al*.* (2019). The large repertoire of conifer NLR resistance genes includes drought responsive and highly diversified RNLs. *Sci. Rep.* 9, 1-3. DOI: 10.1038/s41598-019-47950-7
